# Physiological and proteomic analysis of halophyte *Halogeton glomeratus* in response to Ni^2+^ stress

**DOI:** 10.3389/fpls.2025.1622321

**Published:** 2026-01-30

**Authors:** Lirong Yao, Jianjun He, Juncheng Wang, Baochun Li, Yaxiong Meng, Xiaole Ma, Erjing Si, Hong Zhang, Ke Yang, Huajun Wang

**Affiliations:** 1 State Key Laboratory of Aridland Crop Science, Gansu Key Lab of Crop Improvement and Germplasm Enhancement, Lanzhou, China; 2 Department of Crop Genetics and Breeding, College of Agronomy, Gansu Agricultural University, Lanzhou, China; 3 Department of Botany, College of Life Sciences and Technology, Gansu Agricultural University, Lanzhou, China

**Keywords:** Ni remediation, physiological and molecular mechanisms, differentially abundant proteins, detoxification, biological processes

## Abstract

*Halogeton glomeratus* (*H. glomeratus*) is a halophyte that can remediate heavy metals in soil. However, knowledge regarding the mechanisms of Ni remediation in *H. glomeratus* is limited. In this study, the physiological and molecular mechanisms of *H. glomeratus* seedlings exposed to different Ni^2+^ conditions were investigated. The results revealed that *H. glomeratus* growth was significantly inhibited when the Ni^2+^ concentration was higher than 1.5 mM, but the seedlings did not experience any seedling death and physiological characteristics showed no significant decrease. The accumulation of Ni^2+^ in *H. glomeratus* was found in Ni^2+^-treated seedling roots, stems and leaves. The size of water-storage tissue, the thickness of cortex and the number of large parenchyma cell rose in *H. glomeratus* with the increasing of Ni^2+^ concentrations. Under the 1.5 mmol/L Ni^2+^ for 6 h, 12 h, 24 h, and 48 h, the number of increased abundant proteins was higher than that of decreased abundant proteins at each time point, and numerous differentially abundant proteins mainly involved in response to transmembrane transport, oxidative stress and metabolic process. More importantly, we obtained 36 detoxification-related proteins with increased abundance that were related to Ni^2+^ stress, which were located in apoplast, plasma membrane, vacuolar membrane, chloroplast, and mitochondria, respectively. These biological processes and mechanisms synergistically regulated the Ni^2+^ tolerance in *H. glomeratus*, providing new insights into the application of phytoremediation using wild genetic resources such as halophyte *H. glomeratus.*

## Introduction

Nickel (Ni) pollution represents a significant environmental problem in the contemporary world (Yao et al., 2021). Ni in farmland soil can be absorbed by plants and further enter the food chain. Consuming Ni-contained cereal grains endangers human health, especially in adult females and children (Sun et al., 2013; Müller and Anke, 1994). Ni is the predominant pollutant in farmland soil and the exceedance rate reached 82.8% of all over standard points, indicating a critical status of soil pollution (Hu et al., 2003). Therefore, methods are urgently needed to address the concerns regarding food security and the environment due to Ni contamination. The primary sources of Ni in farmland are human activities including sewage irrigation, pesticide application, mining, and industrial production (Zhuang et al., 2023). Therefore, mitigating Ni soil contamination in mining areas and around industrial facilities is important to control the transfer of Ni into farmland within these regions.

Phytoremediation is a plant-based, environmentally friendly, and low-cost approach to remove contaminants from the environment, and has been advocated for remediating Ni pollution (Liang et al., 2017). Excessive Ni accumulation in plants often results in toxic effects that hinder plant growth and may even cause plant mortality (Oladoye et al., 2022). In previous research, a wide range of hyperaccumulating plants were evaluated for uptake of heavy metals. However, many plants have low Ni tolerance, hindering biomass accumulation and resulting in slow growth rates, such as Ryegrass (*Lolium perenne* L.) and cabbage (*Brassica oleracea* var.*capitata* L.) with sensitivity to Ni toxicity, thereby restricting their use in remediation of Ni-polluted soil (Liang et al., 2017). Norton et al. found that rice shows significant potential for remediation of soil polluted by Ni due to specific characteristics such as high biomass production and Ni tolerance, however, rice tillering is difficult, resulting in a significantly reduced yield (Norton et al., 2008). *Alyssum lesbiacum* showed significant phytoextraction and phytostabilization potential for soils containing Ni, thus, has good potential as a phytoremediation plant for Ni-contaminated soil (Ingle et al., 2005). Alfalfa has a certain adaptability to Ni, however, when the Ni content exceeded 5 mg/L, the seed germination was inhibited and the seed viability index significantly decreased (Zhuang et al., 2023). Phytotoxicity from heavy metals is mainly associated with the generation of reactive oxygen species (ROS) in plants (Andres and Andrea, 2002; Mithofer, 2004). Excessive Ni leads to significant increase in the membrane lipid peroxidation and concentration of hydrogen peroxide (H_2_O_2_) in a few plant species, originating mainly from the NADPH oxidase in the Plasma Membrane (PM) (Rao and Sresty, 2000; Boominathan and Doran, 2002). The plentiful thiol groups present in glutathione (GSH) have the ability to transport a significant quantity of Ni to vacuoles, effectively sequestering it and decreasing the phytotoxic effects associated with Ni (Boominathan and Doran, 2002). In addition, Ni chelation by organic acids leads to a decline in Ni-induced phytotoxicity and contributes to the increase of plant tolerance towards Ni (Pandolfini et al., 1992). Ni in the soil can complex with organic acid and penetrate the symplastic pathways for long-distance Ni translocation (Schickler and Caspi, 2010). Carboxylic acids are more effective than amino acids in mobilizing Ni, and the organic acids contributing to Ni translocation are mainly attributed to the mobility of the organic acid-Ni complex and transpirational pull in plants (Nigam et al., 2001; Salem et al., 1998).

However, many types of plants can absorb Ni from soil but have a small biomass and slow growth, and unable to sufficiently accumulate Ni in Ni-contaminated soil, thus, cannot be widely used to manage Ni-polluted soil (Wang et al., 2014). Halophytes are flora that can survive and reproduce in a salt environment with NaCl concentrations of 200 mM or higher (Flowers and Colmer, 2010; Flowers and Colmer, 2008) and are ideal alternative phytoremediators for removing heavy metals from polluted soil (Liang et al., 2014; Suelee et al., 2017; Tauqeer et al., 2016). Succulent halophytes such as *Suaeda salsa* (Ke-Fu, 1991), *Atriplex nummularia* (Norman et al., 2010), *Kalidium folium* (Zhao et al., 2005), and *Suaeda fruticose* (Chaudhri et al., 1964) are ideally suited to rehabilitate heavy metal-contaminated land by accumulating heavy metals. Furthermore, halophytes have a more efficient antioxidant system and stronger heavy metal stress tolerance than glycophytes including *Tamarix smyrnensis Bunge* (Kadukova et al., 2008), *Mesembryanthemum crystallinum*, and *S. portulacastrum* (Ghnaya et al., 2007).

*Halogeton glomeratus* (*H. glomeratus*) is a succulent halophyte extensively distributed in the arid regions in Northwest China. This species has a strong tolerance to salt stress. In our previous research, *H. glomeratus* seedlings were shown to accumulate 0.17 g/g Na concentration in dry matter of leaves under 500 mM condition for 21 days (Wang et al., 2015). *H. glomeratus* can compartmentalize Na^+^ into vacuoles of succulent leaves and restrict absorption of Na^+^ in the roots in NaCl-affected soils (Yao et al., 2018; Wang et al., 2015). Notably, the contents of Ni^2+^, Cd^2+^, Cu^2+^, Zn^2+^ and Pb^2+^ in seedlings of *H. glomeratus* elevated with the increasing of heavy metal concentrations with or without NaCl addition, and the seed germination, fresh weight, dry weight, radicles relative viability were also higher under the different heavy metals and heavy metal-polluted 100 mM NaCl treatments than that of the control (Yao et al., 2021). A quantity of salts and heavy metals accumulated in *H.glomeratus* growing in the heavy metal-contaminated saline soil plots, and the levels of salt and heavy metals in test soils showed significant decrease compared with unsown plots after sowing *H. glomeratus* seeds in heavy metal contaminated saline soil plots. In addition, *H. glomeratus* had strong heavy meatal Cd resistance, it could accumulate large amounts of Cd^2+^ in leaves, and a lot of detoxification-related differentially abundant proteins with catalytic activities and binding were mainly localized in the plasma membrane, cytoplasm and chloroplast in *H. glomeratus* in response to Cd^2+^ stress (Li et al., 2019; Yao et al., 2022). However, information regarding the molecular mechanisms of heavy meatal Ni remediation in *H. glomeratus* remains unclear. Therefore, in the present study, data-independent acquisition (DIA)-based proteomic experiments were performed in *H. glomeratus* leaves under Ni stress to explore the mechanisms of Ni response and adaption. Furthermore, the Ni hyperaccumulation and detoxification mechanisms of *H. glomeratus*, including Ni stress-regulation genes/proteins, will be further validated. In addition, this study reveals a new role of potential target proteins in regulating Ni tolerance and detoxification in the leaves of halophyte *H. glomeratus*.

## Materials and methods

### Plant materials and Ni stress treatment

The effects of phytoremediation on heavy metal-contaminated saline soils in *H. glomeratus* has been analyzed (Li et al., 2019). In the current research, the calculations on how effective the *H. glomeratus* were to deplete Ni in soil were based on our previous study (Li et al., 2019). Then, *H. glomeratus* seeds were collected in Minqin County of Gansu Province, China (38°01′N, 103°35′E). The collected seeds were sown in plastic pots (diameter, 11 cm; height, 10 cm) filled with sand and vermiculite (1:1 v/v), with 30 seeds per pot. Plants were cultured in the growth chamber under conditions of 25°C/18°C temperature cycle and 16-h light/8-h dark cycle, with irradiation intensity of approximately 300 μmol m^−2^ s^−1^ and 60% relative humidity. Half-strength Hoagland’s solution was used to irrigate the biological samples every day (Wang et al., 2015).

After 1 month, the plants were treated with half-strength Hoagland’s solution with different concentrations (0.0, 0.5, 1.0, 1.5, 2.0, and 3.0 mM) of Ni^2+^ in the form of NiSO_4_ for 20 days (Yao et al., 2021). To avoid Ni^2+^ binding and shock injuries, the Ni^2+^ containing half-strength Hoagland’s solution irrigated in the pallet at the bottom of pot, which could improve the ability of seeding root system to absorb Ni^2+^ and nutrition, and the Ni^2+^ concentrations were increased in 0.5 mM per day increments until the final concentrations (0.5, 1.0, 1.5, 2.0, and 3.0 mM) were achieved. Control plants were cultured in half-strength Hoagland’s solution without Ni^2+^. The Ni^2+^ solutions were changed every day to maintain stable Ni^2+^ concentrations and prevent nutrient deficiency. After Ni^2+^ treatment, different tissues of *H. glomeratus* were collected from control and Ni^2+^-stressed plants for physiological and anatomical analyses. In addition, the seeds were rinsed with distilled water to remove the surface impurities, and then sterilized with 75% alcohol for 1.5 min, the distilled water was treated again to remove the alcohol from the surface of seeds. Each sterilized culture dish was filled with 5 mL of described Ni^2+^ solutions (0.0, 0.5, 1.0, 1.5, 2.0, and 3.0 mM). Finally, the 50 treated seeds were regularly placed in each sterilized culture dishes and germination numbers were recorded at 3 d, 5 d, 7 d and 10 d, respectively (Khan et al., 2001). Three independent experiments were performed as biological replicates.

In a separate experiment, 1-month-old seedlings were stressed with half-strength Hoagland’s solution containing 1.5 mmol/L Ni^2+^ (NiSO_4_) for 0 h, 6 h, 12 h, 24 h, and 48 h, pH was 8.0 (Yao et al., 2021). The control samples were cultured with half-strength Hoagland’s solution without Ni^2+^. Leaves were immediately harvested and maintained at −80°C for proteomic analysis, and three biological replicates were performed at each treatment.

### Tissue microstructure analysis and observation of *H. glomeratus*

Fresh leaves stressed with different Ni^2+^ concentrations were placed on double-sided thick adhesive tape adhered to metal stubs for scanning electron microscopy (SEM; S-3400N, Hitachi Group Company, Tokyo, Japan) of *H. glomeratus* leaf abaxial surfaces under 3.0 kV current and 60 Pa pressure.

In addition, the microstructures were observed in different tissues of Ni-treated *H. glomeratus*. Samples from different Ni treatment conditions were placed in mortars and immediately frozen in liquid nitrogen. The leaf, stem, and root tissues were transferred to a freeze dryer at −55°C for 24 h to remove all water from tissues. Freeze-dried leaf, stems, and roots were cross-cut at approximately 60° angle gradient. Cross-sections were analyzed using SEM as described above. Anatomical features of leaf, stem, and root tissues were treated with the safranin and fast-green stain procedure based on methods described by Schuerger et al (Schuerger et al., 1997).

### Physiological analyses of plants

The percent germination was determined based on the percent of total number of germinated seeds in total number of experimental seeds. The fresh weight (FW) of each sample was calculated immediately after harvesting and the dry weight (DW) was evaluated after drying at 80°C until the weight was stable. Tissue water content (TWC) was measured using the following formula: (FW − DW)/FW × 100%. Each measurement was repeated three times for each treatment condition. The relative root viability was determined using the 2,3,5-triphenyltetrazolium chloride (TTC) reduction method (Juncheng et al., 2016; Munns et al., 2012). Ni^2+^ content was measured as previously described by Munns et al (Munns et al., 1976). Atomic absorption spectrometry (AA240; Varian Medi cal Systems, Palo Alto, CA, USA) was used to analyze Ni^2+^ concentration in the different *H. glomeratus* tissues and growth environments (Li et al., 2019).

In addition, the bioconcentration factor (BF) and transshipment coefficient (TF) were measured using the formulae: BF = Ni^2+^content in root/Ni^2+^ content in the growth environment; TF = Ni^2+^ content in leaf/Ni^2+^ content in the root. The independent replicate consisted of 10 seedling tissues. Three biological replicates were performed for each condition. Significant differences were analyzed using Least Significant Difference (LSD) test with SPSS 19.0 statistical software (SPSS Inc., Chicago, IL, USA, 2021). Mean values in the same column representing the same letters did not significantly differ at *p* < 0.05.

### Protein extraction and enzymatic hydrolysis

Total proteins were extracted from leaves of *H. glomeratus* exposed to Ni^2+^ treatments for different lengths of time (1.5 mmol/L NiSO_4_ for 0 h, 6 h, 12 h, 24 h, and 48 h) based on the method described by Yao et al (Yao et al., 2022). Frozen leaf tissue was suspended in a 5-mm steel bead and appropriate amount of lysis buffer 3 added with 1 mM phenylmethylsulfonyl fluoride (PMSF, final concentration), and 2 mM ethyl-enediaminetetraacetic acid (EDTA, final concentration). Homogenates were vortexed for 5 min. The vortexed pellets were washed with 10 mM dithiothreitol (DTT, final concentration) and oscillated for 2 min (frequency, 50 Hz) using a tissue grinder. The supernatant was collected after centrifugation at 25,000 ×g at 4°C for 20 min and washed again with 10 mM DTT (final concentration) for 1 h at 56°C. Then, the samples were alkylated with 55 mM iodoacetamide (final concentration) for 45 min in a dark room at 25°C. The mixtures were precipitated by a 4× volume of cold acetone for 2 h at −20°C until the supernatant was colorless. After centrifugation at 25,000 ×g for 20 min at 4°C, the pellets were precipitated with lysis buffer 3, followed by ultrasonication to dissolve the precipitated proteins. The supernatant proteins were obtained for quantification after centrifugation at 25,000 ×g at 4°C for 20 min and the protein concentration evaluated using the Bradford method with bovine serum albumin as the standard (Bradford, 1976).

For protein enzymatic hydrolysis, the collected proteins (100 μg per sample) were diluted with 4× volume of NH_4_HCO_3_ (50 mM). The diluted proteins were digested in 2.5 μg trypsin enzyme (v:v, protein:enzyme, 40:1) for 4 h at 37°C. Next, another digest was performed using the same ratio at 37°C for 8 h to digest proteins. Finally, enzymatic peptides were desalted in a Strata X column and vacuum-dried. All experiments were performed in triplicate.

### Peptide separation and LC-MS/MS analysis

The complex peptides were fractionated using a Shimadzu LC-20AB HPLC pump system (Shimadzu, Kyoto, Japan) combined with a Gemini high pH C18 column (5 μm, 250 × 4.6 mm). The 10 μg of each sample was blended and the 200 μg mixture was diluted using 2 mL of mobile phase A (5% acetonitrile (CAN), pH 9.8). The diluted mixture was injected into the column and purged with 5% mobile phase B (95% CAN, pH 9.8) for 10 min, followed by mobile phase B (concentration from 5% to 35%) for 40 min, then 35% to 95% mobile phase B for 1 min; phase B flow lasted 3 min followed by 5% mobile phase B equilibration for 10 minutes. Peptide fractions were obtained by integrating components with an elution peak at 214 nm at every time point and then freeze-dried.

MS-based proteomics, as previously described by Cox and Mann (Jürgen and Matthias, 2008), can efficiently and robustly extract information from raw MS data with high protein quantification accuracy and peptide identification rate for analysis (Jürgen and Matthias, 2008). The DIA approach has recently been considered a novel MS method that combines shotgun proteomics with the reproducibility and precision of selected reaction monitoring and uses iRT peptides for retention time (Bruderer et al., 2015). In the present study, the DDA fractions and DIA analysis were performed using a Q Exactive HF mass spectrometer combined with an Ultimate 3000 RSLCnano system (Thermo Fisher Scientific, Waltham, MA, USA) (Bruderer et al., 2015; Jürgen and Matthias, 2008).

### Protein quantification and database searching

The *H. glomeratus* ISO-Seq transcriptome database (NCBI; BioProject ID: PRJNA 359784) *via* MaxQuant software (http://www.maxquant.org) was used for protein identification and quantification. A non-redundant high-quality MS/MS spectrogram was used as the spectrogram library for subsequent DIA quantification. To decrease the false peptide identification probability, the peptides with < 95% confidence interval (CI; p < 0.05) containing a false discovery rate ≤ 1% were chosen to build the final spectral library. Each successfully identified protein possessed at least one credible unique peptide.

For protein quantitation estimation, MSstats was used to statistically estimate differentially abundant proteins/peptides based on linear mixed-effects; the control group was considered a reference to determine the statistical significance based on the model (Meena et al., 2014). Ratios with an absolute fold change ≥ 1.5 and p-value < 0.05 were used to analyze the significant differentially abundant proteins. To determine their biological processes, the agriGO (http://bioinfo.cau.edu.cn/agriGO/index.php) tool was used to identify the significantly enriched Gene Ontology (GO) terms related to these proteins, and Kyoto Encyclopedia of Genes and Genomes database (KEGG) pathway analysis were also utilized to annotate functional information about these assembled proteins. In addition, the differentially abundant proteins were detected using a Venn diagram (http://bioinfogp.cnb.csic.es/tools/venny/index.html) for plants treated with 1.5 mM Ni^2+^ for 0 h, 6 h, 12 h, 24 h, and 48 h, and the common differentially abundant proteins were identified using Hierarchical Cluster (https://www.omicshare.com/tools/Home/Soft/heatmap). The functions and metabolomics of differentially abundant proteins were categorized according to the Bevan et al. scheme (Bevan et al., 1998).

### Identification of differentially abundant proteins based on Multiple Reaction Monitoring (MRM)

To identify the reliability of proteomics data, multiple reaction monitoring (MRM) analysis was further used to validate the key preferred biomarker (Chen et al., 2020). MRM analysis was performed using a QTRAP5500 MS (AB SCIEX, Foster City, CA, USA) with the LC-20 CE nanoHPLC system (Shimadzu, Kyoto, Japan) as described by Chen et al (Chen et al., 2020). Six differentially abundant proteins were randomly chosen for MRM research of plants treated with 1.5 mmol/L Ni^2+^ for 0 h, 12 h, 24 h, and 48 h. All experiments were performed in triplicate.

## Results

### Characteristics of *H. glomeratus* Ni^2+^ absorption

**Figure 1:**
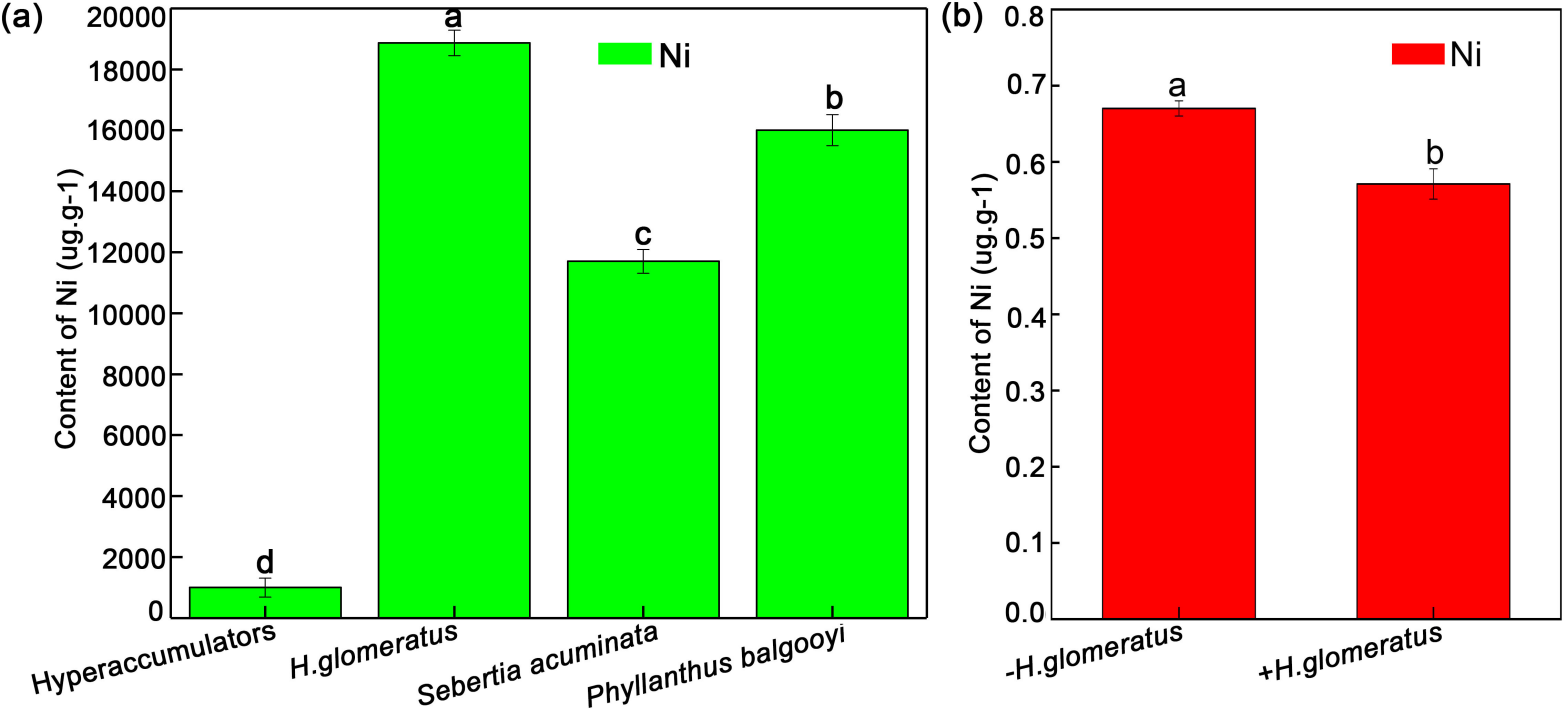
Comparison of Ni^2+^ accumulation between hyperaccumulators and *H. glomeratus***(a)** and Ni^2+^ content in soil sown with *H. glomeratus* and unsown *H. glomeratus***(b)**. Three independent replicates were performed for each experiment (n = 3). Mean values in the same column with the same letters did not significantly differ at p < 0.05.

According to the previous definition, the proposed nominal Ni threshold criteria for hyperaccumulators was 1,000 ug/g (in units of µg metal per g of dry leaf tissue) (Liang et al., 2017); The content of Ni was 11,700μg/g (in units of µg metal per g of dry leaf tissue) in leaves of hyperaccumulators *Sebertia acuminata* growing in heavy metal contaminated soil (**Figure 1a**) (Jaffré et al., 1976), and hyperaccumulators *Phyllanthus balgooyi* could accumulate 16,000 μg/g (in units of µg metal per g of dry leaf tissue) Ni in leaves under same environment condition (**Figure 1a**) (Hoffmann et al., 2003). The Ni accumulation in *H. glomeratus* leaves was 18,870 ug/g (in units of µg metal per g of dry leaf tissue), an increase of 17.87-fold compared with the threshold criteria (**Figure 1a**) (Li et al., 2019). However, the concentration of Ni at the 0–20 cm depth in soil from Minqin (-*H. glomeratus*) was 0.67 mM, which decreased by 14.81% (0.57 mM) in *H. glomeratus* in a single growing season (p < 0.05; **Figure 1b**) (+*H. glomeratus*) (Li et al., 2019). Thus, *H. glomeratus* was considered as the hyperaccumulators and confirmed to remove the Ni from heavy metal-polluted soil, effectively preventing the accumulation of Ni in the topsoil and tolerating Ni stress.

**Figure 2:**
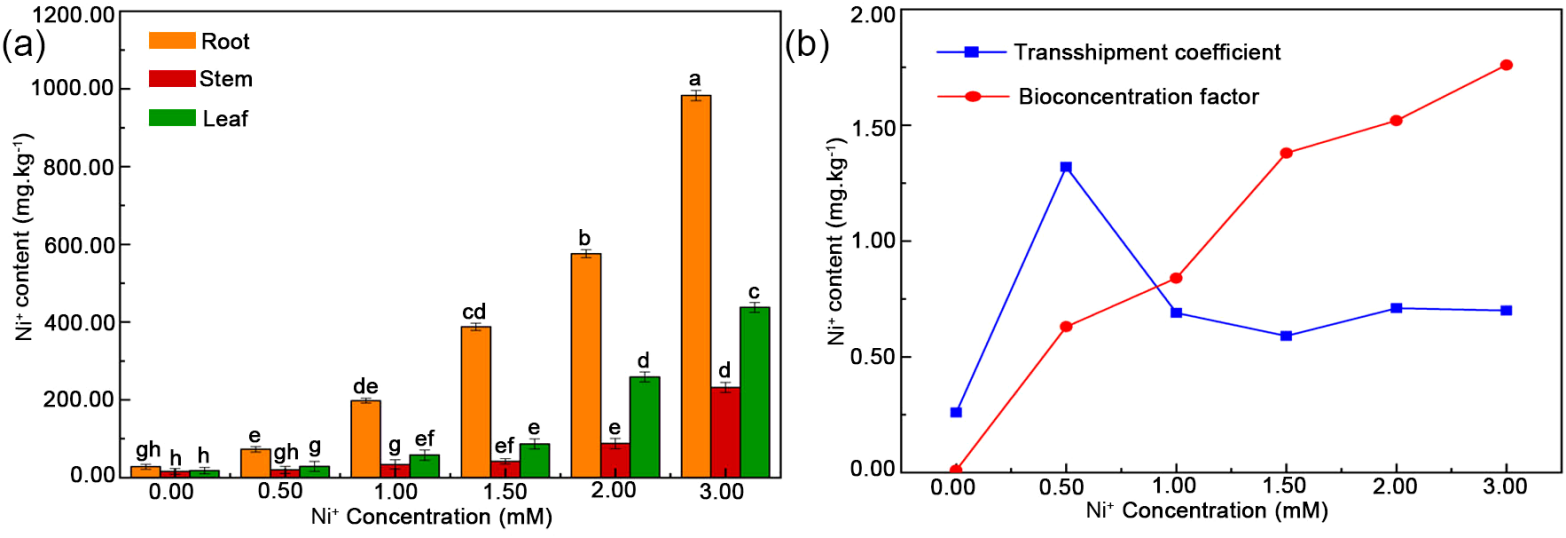
Effects of different Ni^2+^ concentrations on plant properties. **(a)** Effects of increasing Ni^2+^ concentrations on the ion content in leaves, stems, and roots of *H. glomeratus*. **(b)** Effects of increasing Ni^2+^ concentrations on the bioconcentration factor and the transshipment coefficient. Three independent replicates were performed for each experiment (n = 3). Mean values in the same column with the same letters did not significantly differ at p < 0.05.

Compared with control samples, the Ni^2+^ contents increased in the leaves, stems, and roots of plants treated with 0.0, 0.5, 1.0, 1.5, 2.0, and 3.0 mM Ni^2+^ for 20 days. This increase was evident at 1.5 mM Ni^2+^, reaching 80.50 mg/kg in leaves, 29.30 mg/kg in stems, and 380.30 mg/kg in roots (**Figure 2a**). However, the Ni^2+^ contents in different tissues of *H. glomeratus* at the same concentration of Ni^2+^ were significantly different (p < 0.05); highest in roots followed by leaves and then stems (**Figure 2a**). Under 3.0 mM Ni^2+^ condition, Ni^2+^ contents were 236.10 mg/kg in leaves, 467.20 mg/kg in stems, and 967.10 mg/kg in roots. Thus, the Ni^2+^ contents were 2.07-fold and 4.10-fold higher in roots than in stems and leaves, respectively. In addition, the TF first increased and then decreased with increasing Ni^2+^ concentrations, with a maximum value of 1.34 under 0.5 mM Ni^2+^ condition, and the BF markedly increased under Ni^2+^ stress (**Figure 2b**). Thus, these results affirmed *H. glomeratus* seedlings can accumulate Ni^2+^ from roots and transfer it into the shoots, indicating high potential value of *H. glomeratus* for Ni^2+^ phytoremediation.

### Effects of Ni concentration on *H. glomeratus* seedling growth

**Figure 3:**
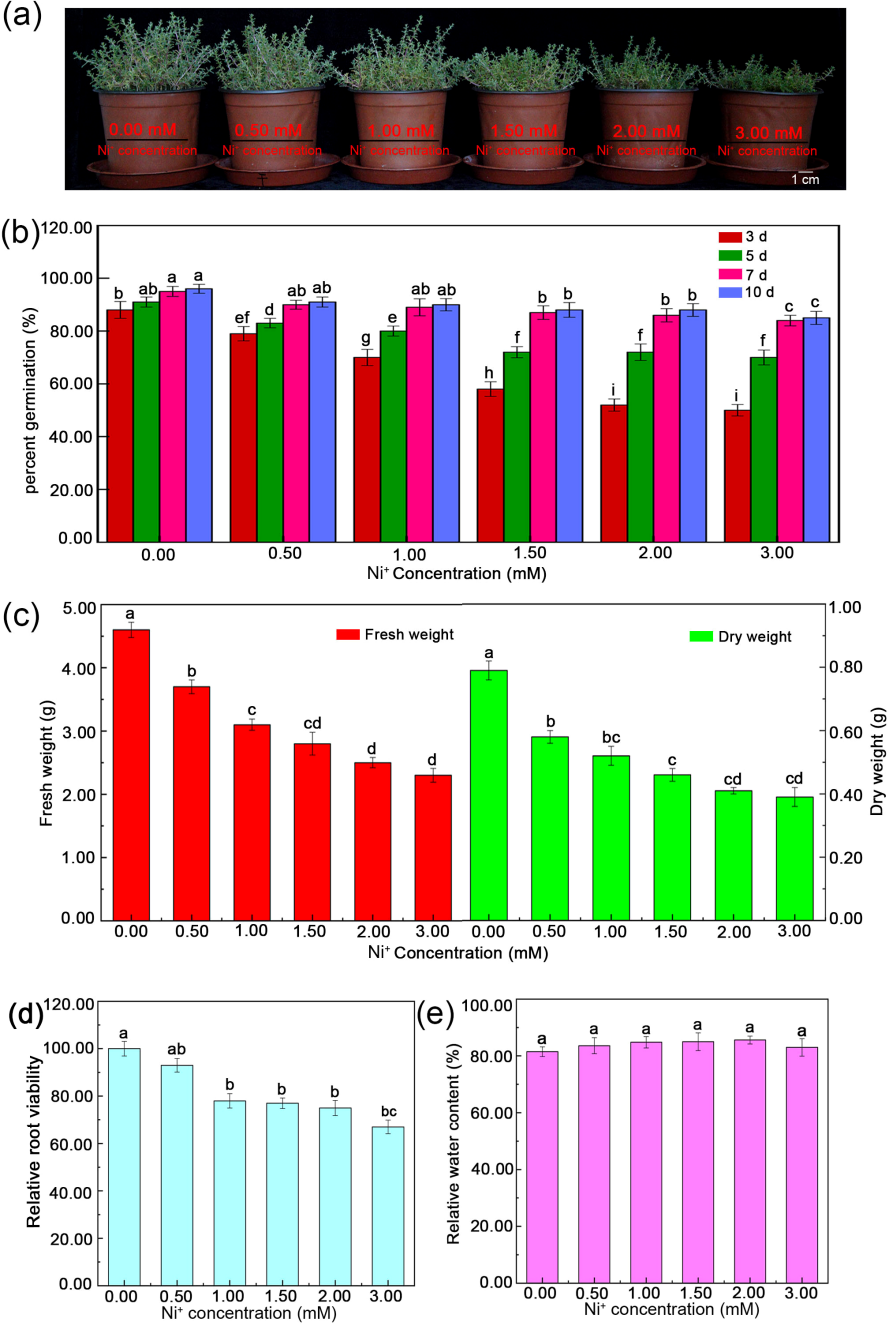
Effects of Ni^2+^ stress on the growth, percent germination, fresh weight, dry weight, relative root variety, and relative water content of *H. glomeratus*. **(a)** The plants were treated with half-strength Hoagland’s solution containing different concentrations (0.0, 0.5, 1.0, 1.5, 2.0, or 3.0 mM) of Ni^2+^ for 20 days. **(b)** Effects of increasing Ni^2+^ concentrations on the germination percentage of *H. glomeratus* seeds. **(c)** Effects of increasing Ni^2+^ concentrations on the fresh weight and dry weight of *H. glomeratus*. **(d)** Effects of increasing Ni^2+^ concentrations on the relative root viability of *H. glomeratus*. **(e)** Effects of increasing Ni^2+^ concentrations on the relative water content of *H. glomeratus.* Three independent replicates were performed for each experiment (n = 3). Mean values in the same column with the same letters did not significantly differ at p < 0.05.

Plants grew well under different concentrations of Ni. Compared with control, higher Ni concentrations (1.50-3.00 mM) significantly inhibited seedling growth (**Figure 3a**). The germination rate of *H. glomeratus* decreased at first and then remained stable with increasing Ni treatment. The Ni^2+^ concentrations > 1.5 mM significantly decreased the rate of germination (**Figure 3b**). Seedling FW, DW, and relative root viability of *H. glomeratus* also decreased at first and then remained unchanged but significantly decreased when the Ni concentration was > 1.5 mM (**Figure 3c**). In particular, at 3.00 mM Ni concentration, relative root viability reached 63.28% and the *H. glomeratus* seedlings did not die (**Figure 3d**). The TWC in leaves slightly increased first and then plateaued with the increasing of Ni concentrations (**Figure 3e**). Which indicated that *H. glomeratus* could survive at 3.00 mM Ni level and did not experience any seedling death.

### Anatomical structure and SEM analyses of *H. glomeratus* tissues

**Figure 4:**
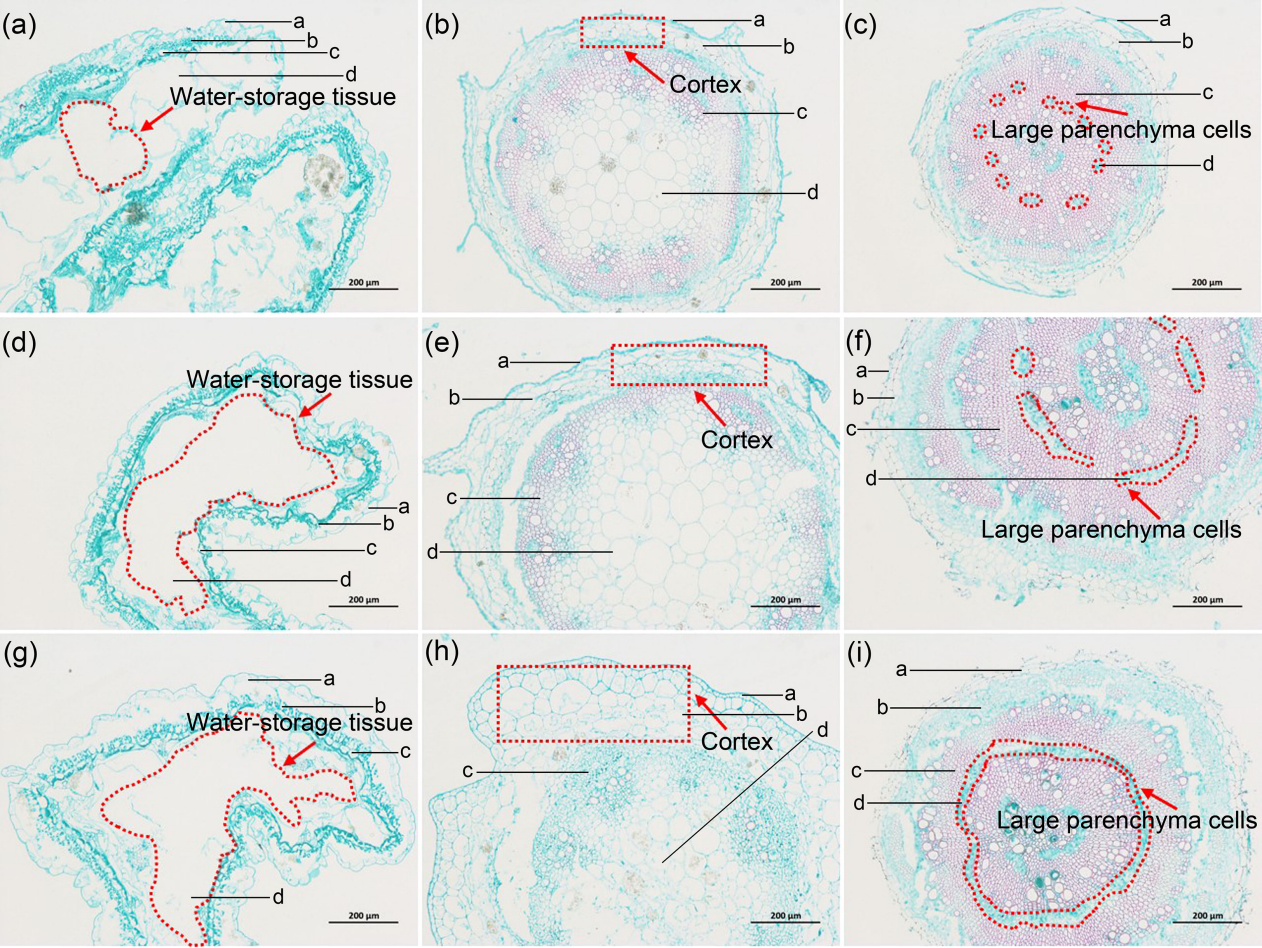
Transverse sections of leaves, stems, and roots of *H. glomeratus* seedlings treated with different Ni^2+^ concentrations. **(a)** Transverse sections of leaves of *H. glomeratus* under 0.0 mM Ni^2+^ treatment. a, epidermal cells. b, palisade tissue. c, lignified cells. d, water-storage tissue. **(b)** Transverse sections of stems of *H. glomeratus* under 0.0 mM Ni^2+^ treatment. a, epidermal cells. b, cortex. c, hollow pith. d, collateral vascular bundle. **(c)** Transverse sections of roots of *H. glomeratus* under 0.0 mM Ni^2+^ treatment. a, cortex. b, secondary phloem. c, xylem. d, large parenchyma cells. **(d)** Transverse sections of leaves of *H. glomeratus* under 1.5 mM Ni^2+^ treatment. a, epidermal cells. b, palisade tissue. c, lignified cells. d, water-storage tissue. **(e)** Transverse sections of stems of *H. glomeratus* under 1.5 mM Ni^2+^ treatment. a, epidermal cells. b, cortex. c, hollow pith. d, collateral vascular bundle. **(f)** Transverse sections of roots of *H. glomeratus* under 1.5 mM Ni^2+^ treatment. a, cortex. b, secondary phloem. c, xylem. d, large parenchyma cells. **(g)** Transverse sections of leaves of *H. glomeratus* under 3.0 mM Ni^2+^ treatment. a, epidermal cells. b, palisade tissue. c, lignified cells. d, water-storage tissue. **(h)** Transverse sections of stems of *H. glomeratus* under 3.0 mM Ni^2+^ treatment. a, epidermal cells. b, cortex. c, hollow pith. d, collateral vascular bundle. **(i)** Transverse sections of roots of *H. glomeratus* under 3.0 mM Ni^2+^ treatment. a, cortex. b, secondary phloem. c, xylem. d, large parenchyma cells. The ruler is 200 um.

Based on the cross-sectional analysis of *H. glomeratus* tissues under different Ni^2+^ concentrations, successive cambia phenomenon existed in the stems and roots and water-storage tissue was present in leaves (**Figure 4**). Notably, the size of water-storage tissue in leaves, the thickness of cortex in stems, and the number of large parenchyma cells in *H. glomeratus* roots increased with increasing Ni^2+^ concentration.

**Figure 5:**
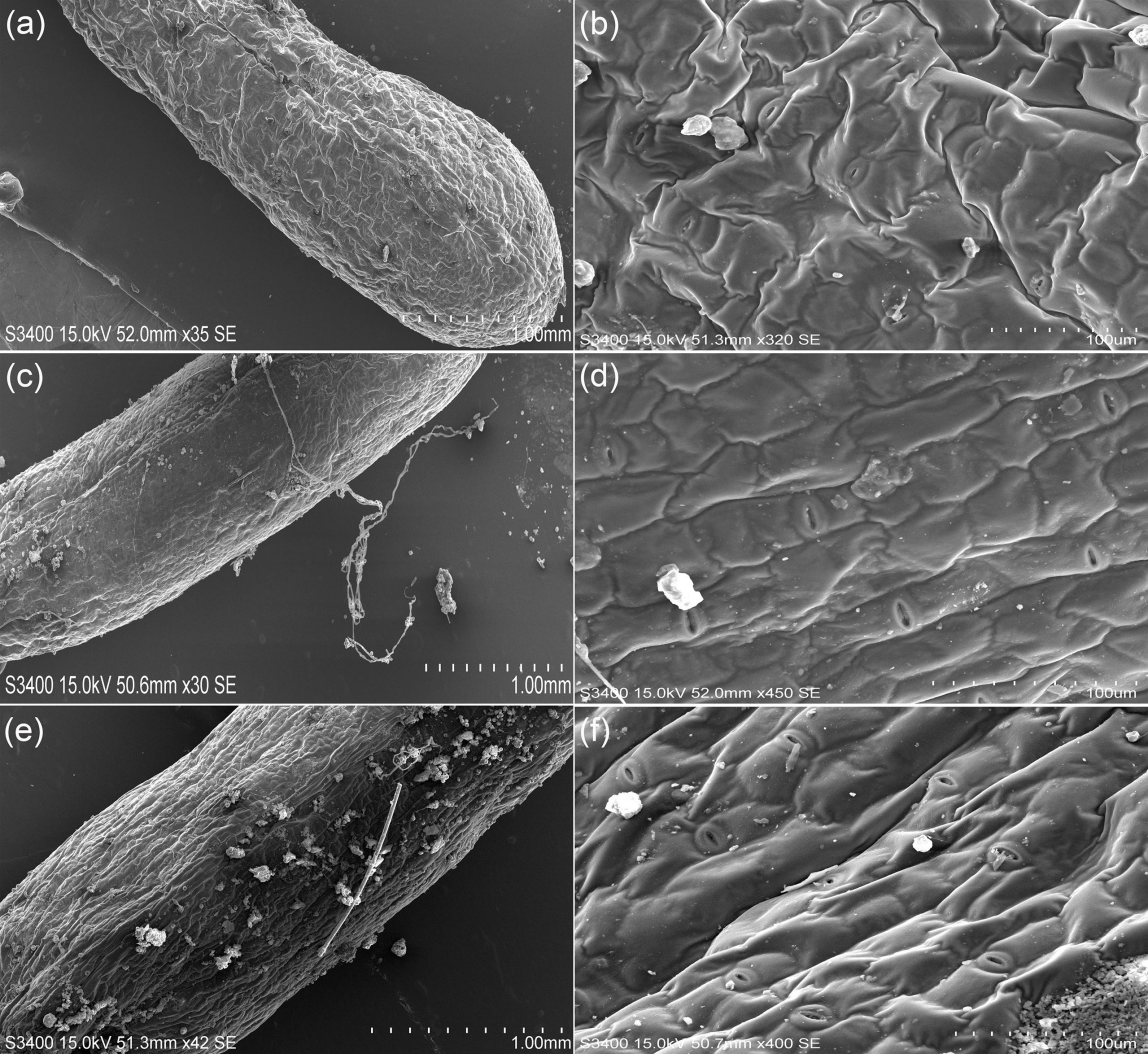
Micrographs of leaf surfaces of *H. glomeratus* seedlings treated with different concentrations of Ni^2+^. **(a)** Micrograph of leaf surfaces of *H. glomeratus* seedlings under 0.0 mM Ni^2+^ treatment. **(b)** Partial enlargement of leaf surfaces of *H. glomeratus* seedlings under 0.0 mM Ni^2+^ treatment. **(c)** Micrograph of leaf surfaces of *H. glomeratus* seedlings under 1.5 mM Ni^2+^ treatment. **(d)** Partial enlargement of leaf surfaces of *H. glomeratus* seedlings under 1.5 mM Ni^2+^ treatment. **(e)** Micrograph of leaf surfaces of *H. glomeratus* seedlings under 3.0 mM Ni^2+^ treatment. **(f)** Partial enlargement of leaf surfaces of *H. glomeratus* seedlings under 3.0 mM Ni^2+^ treatment.

**Figure 6:**
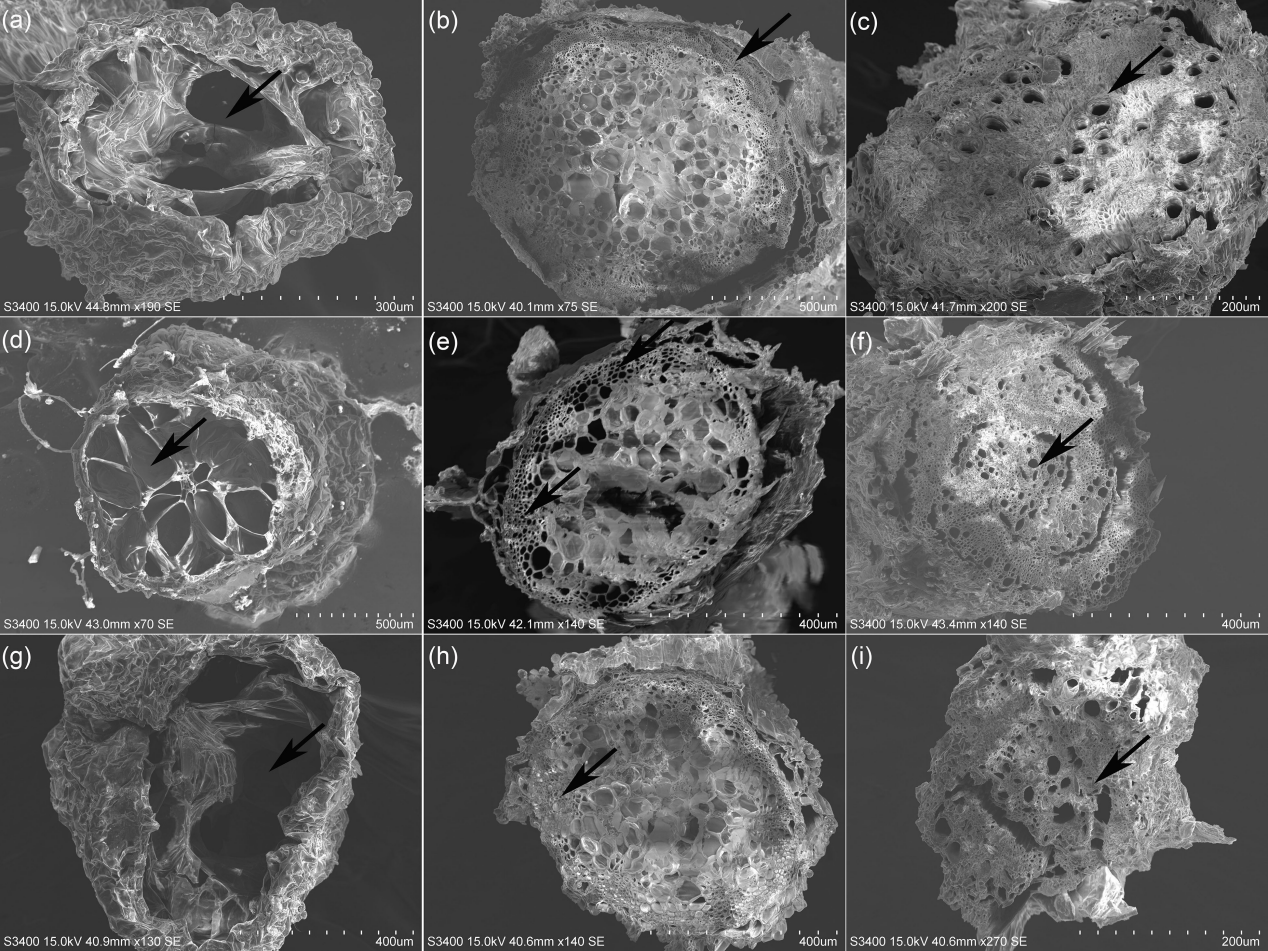
Structure of leaves, stems, and roots of *H. glomeratus* seedlings treated with different concentrations of Ni^2+^. **(a)** Leaf structure of *H. glomeratus* under 0.0 mM Ni^2+^ treatment. **(b)** Stem structure of *H. glomeratus* under 0.0 mM Ni^2+^ treatment. **(c)** Root structure of *H. glomeratus* under 0.0 mM Ni^2+^ treatment. **(d)** Leaf structure of *H. glomeratus* under 1.5 mM Ni^2+^ treatment. **(e)** Stem structure of *H. glomeratus* under 1.5 mM Ni^2+^ treatment. **(f)** Root structure of *H. glomeratus* under 1.5 mM Ni^2+^ treatment. **(g)** Leaf structure of *H. glomeratus* under 3.0 mM Ni^2+^ treatment. **(h)** Stem structure of *H. glomeratus* under 3.0 mM Ni^2+^ treatment. **(i)** Root structure of *H. glomeratus* under 3.0 mM Ni^2+^ treatment. Arrows indicate water-storage tissue in leaves, cortex in stems, and large parenchyma cells in root.

After seedlings were treated with different concentrations (0, 1.5, or 3.0 mM) of Ni^2+^ for 72 h, the SEM study of leaf abaxial surfaces showed Ni was not secreted to the leaf surface and the size and density of stomata differed (**Figure 5**). The SEM micrographs of the tissue cross-sections showed the size of leaf water-storage tissues, the thickness of stem cortices, and the number of root large parenchyma cells in *H. glomeratus* treated under different Ni^2+^ conditions were higher than in control treatment, similar to what was observed at the anatomical structure level, especially at higher Ni^2+^ concentrations (**Figure 6**).

### Changes of protein profiles in leaves under Ni^2+^ stress

**Figure 7:**
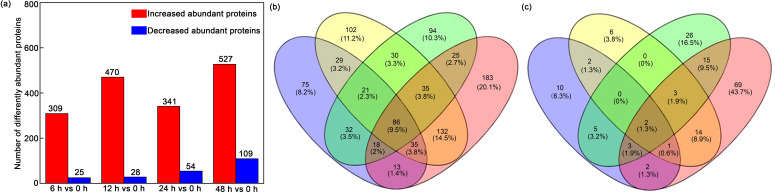
The number and differentially abundant proteins based on proteomics of *H. glomeratus* in response to Ni^2+^ stress. **(a)** Increased-abundant and reduced-abundant proteins based on proteomics of *H. glomeratus* treated with different Ni^2+^ concentrations. **(b)** Venn diagram showed the increased abundant proteins under 1.5 mM Ni^2+^ treatment for 6, 12, 24. and 48 h. **(c)** Venn diagram showed the decreased abundant proteins under 1.5 mM Ni^2+^ treatment for 6, 12, 24. and 48 h. Three independent replicates were performed for each experiment (n = 3).

To examine proteomic changes in the *H. glomeratus* leaves in response to Ni^2+^ stress, the protein profiles of leaves exposed to 1.5 mM Ni^2+^ for 0 h, 6 h, 12 h, 24 h, and 48 h were evaluated using a DIA proteomic method. A total of 25,958 peptides and 4,795 proteins were found (**Supplementary Files 1**, **2**). As shown in **Supplementary Files 3**, only 5.88% of the proteins were matched with > 11 unique peptides, and the protein numbers decreased with the increasing number of unique peptide segments. Proteins that had two unique peptides (1,385, 21.72%) were quantified based on the 95% CI (p < 0.05) and a 1.5-fold change for significant difference. Compared with untreated control samples, 309, 470, 341, and 527 differentially abundant proteins with increased abundance (significantly increased abundant proteins) were identified, and 25, 28, 54, and 109 proteins showed reduced abundance (significantly reduced abundant proteins) after seedlings were exposed to 1.5 mM Ni^2+^ for 6, 12, 24, and 48 h, respectively (**Figure 7**, **Supplementary Files 4**-**7**), which were considered to be the dynamic process to Ni^2+^ stress for *H. glomeratus*. The number of increased abundant proteins was significantly higher than of reduced abundant proteins.

**Figure 8:**
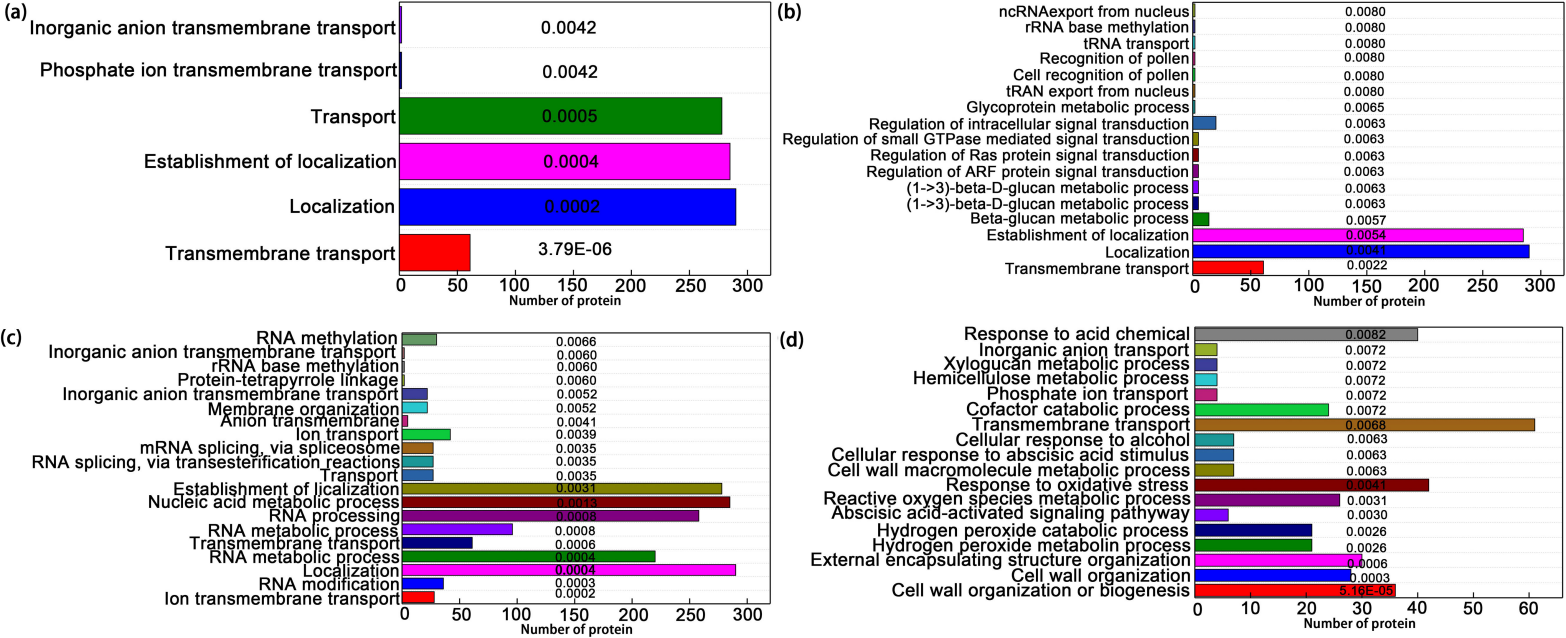
Differentially abundant proteins involved in primary biological processes in *H. glomeratus* based on GO classification. **(a)***H. glomeratus* plants treated with 1.5 mM Ni^2+^ for 6 h. **(b)***H. glomeratus* plants treated with 1.5 mM Ni^2+^ for 12 h. **(c)***H. glomeratus* plants treated with 1.5 mM Ni^2+^ for 24 h. **(d)***H. glomeratus* plants treated with 1.5 mM Ni^2+^ for 48 h. Each column represents a biological process and the numerical values represent p-value of each biological process.

The biological processes of the identified differentially abundant proteins annotated based on the GO annotation and enrichment analysis are shown in **Figure 8** and **Supplementary Files 8**-**11**. At the early stage (6 h) of Ni^2+^ stress, the dominant biological processes were “transmembrane transport”, “localization”, “establishment of localization”, “transport”, “phosphate ion transmembrane transport”, and “inorganic anion transmembrane transport” (**Figure 8a**). At the middle stages (12 and 24 h) of Ni^2+^ stress, the prevalent categories of biological processes included “transmembrane transport”, “localization”, “establishment of location”, “nucleic acid metabolic process”, “beta-glucan metabolic process”, “glycoprotein metabolic process”, and “regulation of intracellular signal transduction” (**Figures 8b, c**). However, at the later stage (48 h) of Ni^2+^ stress, the dominant terms were “cell wall organization or biogenesis”, “external encapsulating structure organization”, “hydrogen peroxide metabolic process”, “hydrogen peroxide catabolic process”, “response to oxidative stress”, “cellular response to abscisic acid stimulus”, “cellular response to alcohol”, and “response to acid chemical” (**Figure 8d**). These changes offer key information regarding dynamic proteomic alterations in research on Ni-tolerant *H. glomeratus* leaves.

We obtained KEGG pathway annotation for these differentially abundant proteins. The pathways including the “Cellular processes”, “Environmental information processing”, “Genetic information processing”, “Human diseases”, “Metabolism” and “Organismal systems” with Q values ≤ 0.05 were found to be significant enrichment (**Supplementary File 12**). A total of 256, 379, 312 and 564 differentially abundant proteins assigned to these pathways in the Ni^2+^-treated samples at 6, 12, 24, and 48 h, respectively (**Supplementary File 13**-**16**). The enrichment pathways with more proteins than the other pathways were “Metabolism” and “Genetic information processing”, which contains “Carbohydrate metabolism”, “Energy metabolism”, “Biosynthesis of other secondary metabolites”, “Folding, sorting and degradation”, “Translation” and “Transcription”. However, the significant enrichment pathways of “Transport and catabolism”, “Membrane transport”, “Signal transduction” and “Environmental adaptation” were also detected for *H. glomeratus* under Ni^2+^ treatments. These annotations are a valuable resource for Ni tolerance in *H. glomeratus* research.

### Proteins potentially regulated by Ni^2+^ stress and detoxification

**Figure 9:**
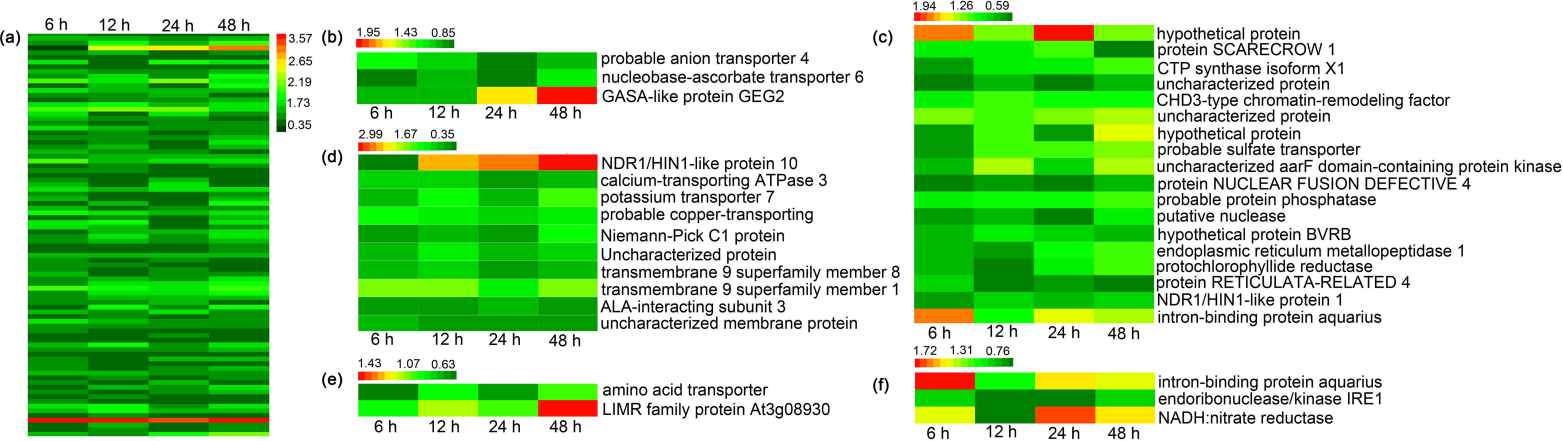
Hierarchical clustering and functional cataloging of differentially highly abundant proteins in *H. glomeratus* under Ni^2+^ stress conditions. **(a)** Hierarchical clustering of the common differentially highly abundant proteins with 1.5 mM Ni^2+^ for 6, 12, 24, and 48 h. **(b)** Functional cataloging of the common differentially highly abundant proteins in the apoplast according to the Bevan et al. scheme (Bevan et al., 1998). **(c)** Functional cataloging of the common differentially highly abundant proteins in the chloroplast according to the Bevan et al. scheme (Bevan et al., 1998). **(d)** Functional cataloging of the common differentially highly abundant proteins in the plasma membrane according to the Bevan et al. scheme (Bevan et al., 1998). **(e)** Functional cataloging of the common differentially highly abundant proteins in the vacuolar membrane according to the Bevan et al. scheme (Bevan et al., 1998). **(f)** Functional cataloging of the common differentially highly abundant proteins in the mitochondria according to the Bevan et al. scheme (Bevan et al., 1998). A bar chart to visually display the biological pathways involved in protein expression, with p-value levels annotated.

The present study identified proteins associated with the mechanisms of Ni^2+^ tolerance in *H. glomeratus.* A total of 86 differentially increased abundant proteins were common to various stages of Ni^2+^ stress (**Figure 7b**, **Supplementary File 17**). The protein abundant patterns are shown in **Figure 9b** and were mainly located in plasma membrane, vacuolar membrane, mitochondria, chloroplast, and cytosol. These differentially abundant proteins were associated with the following: probable anion transporter 4, nucleobase-ascorbate transporter 6, and GASA-like protein GEG2 located in the cytosol; NDR1/HINK-like protein 10, Ca-transporting ATPase 3, potassium transporter 7, probable copper-transporting, Niemann-Pick C1 protein, uncharacterized protein, transmembrane 9 superfamily member 8, transmembrane 9 superfamily member 1, ALA-interacting subunit 3, and uncharacterized membrane protein located in the plasma membrane; amino acid transporter and LIMR family protein at3g08930 located in the vacuolar membrane; hypothetical protein, protein SCARECROW 1, CTP synthase isoform X1, probable sulfate transporter, probable protein phosphatase, putative nuclease, endoplasmic reticulum metallopeptidase 1, protochlorophyllide reductase, and intron-binding protein aquarius located in the chloroplast; intron-binding protein aquarius, endoribonuclease/kinase IRE1 and NADH: nitrate reductase located in the mitochondria (**Figure 9**, **Supplementary File 18**). These commonly expressed proteins may play an important role in Ni detoxification for *H. glomeratus*.

### MRM verification analysis for differentially abundant proteins

MRM verification of 6 differentially abundant proteins was performed in *H. glomeratus* seedlings treated with 1.5 mM Ni^2+^ for 0, 12, and 48 h. In these treatment stages, the abundance trends of the 6 differentially abundant proteins based on MRM analyses were roughly consistent with the DIA analysis (**Table 1**). These results indicated the proteomics profiling data can show how *H. glomeratus* responds to Ni^2+^ stress for phytoremediation.

**Table 1:** Proteins associated with multiple reaction monitoring (MRM) verification.

Protein ID	Fold change of 12 h/0 h	Adjusted p-value	Fold change of 48 h/0 h	Adjusted p-value
*Hg.32505*	-1.656	<0.0001	-1.21	<0.0001
*Hg. 47599*	-0.634	<0.0001	-0.69	0.002
*Hg.10630*	1.084	0.017	1.09	0.0087
*Hg.11933*	0.749	<0.0001	1.38	<0.0001
*Hg.35509*	0.874	0.031	0.904	0.028
*Hg.37251*	1.198	0.001	0.949	0.002

## Discussion

Halophyte *H. glomeratus* had robust resistance to heavy metal Cd stress, could accumulate large amounts of Cd in leaves, it also found that *H. glomeratus* had special tolerance mechanisms for Cd^2+^ stress by reducing or avoiding Cd^2+^ toxicity (Yao et al., 2022). *H. glomeratus* seedlings could survive in the heavy metals and heavy metals combined with salinity stress, which also potentially accumulated a lot of heavy metals (Cu^2+^, Ni^2+^, Zn^2+^, Cd^2+^ and Pb^2+^) from the soil, and the levels of heavy metals in test soils were significantly decreased after sowing *H. glomeratus* seeds in heavy metal contaminated saline soil plots compared with unsown plots, especially heavy metal Ni in soil (Yao et al., 2021; Li et al., 2019). Ni stress can induce ROS accumulation in a plant, causing a chain reaction of residue peroxidation that modifies amino acids and affects the structure of proteins (Berlett and Stadtman, 1997). Enhanced plant Ni resistance can aid in achieving Ni absorption and accumulation over prolonged periods. Increasing the understanding of the mechanisms that regulate Ni accumulation in *H. glomeratus* leaves will facilitate the development of strategies for easy removal of accumulated Ni in the contaminated soil in Minqin County of Gansu Province, decreased from 0.67 mM to 0.57 mM (**Figure 1**). However, the molecular mechanisms contributing to Ni resistance and accumulation remain unclear in *H. glomeratus.* In the present study, the morphological, physiological, and proteomics analyses of *H. glomeratus* were integrated to comprehensively understand the distinct responses to Ni toxicity and accumulation strategies.

Phytoremediation is the process in which plants remove pollutants from the environment and has attracted widespread attention in current years because it is a cost-effective and environmentally friendly method (Lü et al., 2013). Heavy metals induce both oxidative stress and secondary water stress in plants, and halophytes can synthesize organically compatible solutes to tolerate heavy metals (Manousaki and Kalogerakis, 2011; Nedjimi and Daoud, 2009; Fürst et al., 1988). To date, researchers have performed numerous studies on phytoremediation of heavy metal-contaminated soils using halophytes (Christofilopoulos et al., 2016; Korzeniowska and Stanisawska-Glubiak, 2015). In the present study, halophyte *H. glomeratus* accumulated significant amounts of Ni within one growing season; 18870 ug/g of Ni accumulated in *H. glomeratus* from heavy metal-polluted saline soils, reaching the normal threshold for hyperaccumulators (**Figures 1a, b**). In addition, the Ni level was only 0.57 mM after *H. glomeratus* was harvested at the end of the growing season, and decreased 14.81% (33.45 mg/kg; **Figure 1b**). However, the overall range of Ni^2+^ concentration in polluted soil was 10–1,000 mg/kg (Li et al., 2005). Thus, continuous cropping of *H. glomeratus* could accumulate Ni^2+^, resulting in decreased Ni^2+^ concentration (< 10 mg/kg) in the soil. These results indicated *H. glomeratus* biomass can accumulate Ni and its harvest process helps to significantly reduce Ni concentration in soils.

Belowground plant tissues are generally the key sink area for most heavy metals, accounting for a large amount of the total phytoaccumulated metal (Anjum et al., 2014). Several halophytes can accumulate Ni from the soil environment, and the accumulation is higher in roots than shoots and leaves. In addition, the highest BFs and lowest translocation factors in plants have the greatest potential for phytostabilization (Kachout et al., 2012). *Atriplex undulata* and *Atriplex lentiformis* decreased heavy metal accumulation in their aerial parts and could be used for phytostabilization of heavy metals (Eissa, 2015). Cambrolle et al. (2008) found that *Spartina maritima* and *Spartina densiflora* accumulated heavy metals in both aboveground and belowground tissues and were highly effective for the phytostabilization of soils (Cambrolle et al., 2008). However, in the present study, Ni^2+^ accumulated in the leaves, stems, and roots of *H. glomeratus* under different Ni^2+^ concentrations, with the highest Ni^2+^ contents found in roots when plants were grown in the presence of 3.0 mM Ni^2+^ (**Figure 2a**). The levels increased from stems to leaves to roots, in that order, and the BF increased with increasing Ni^2+^ concentration (**Figure 2b**). However, there is a second parameter for natural metal hyperaccumulation, namely a shoot:root ratio>1 for the metal concerned (Boominathan and Doran, 2002). This criterion is not met by *H. glomeratus* where the ratio is approximately 0.2 (**Figures 2a, b**), future prospective studies are needed to explain this phenomenon in Ni^2+^ hyperaccumulation of *H. glomeratus*.

*H. glomeratus* is a specialized halophyte with well-adapted morphological, physiological, and anatomical characteristics that allow the plants to survive in a soil environment with a high salt concentration (Wang et al., 2015). Similarly, these features could be found in *H. glomeratus* grown under different Ni concentration conditions. The results of the present study showed *H. glomeratus* growth was affected by Ni concentrations, especially the Ni concentrations were > 1.5 mM (**Figure 3a**). Reportedly, for many halophytes, such as *Atriplex rosea*, *Atriplex hortensis* var. *rubra*, and *Atriplex hortensis* var. *purpurea*, plant height was decreased under high Ni concentration conditions and stimulated under moderate Ni concentration conditions (Kachout et al., 2012). The effects of different Ni concentrations (0 and 0.05 mM) on seedling size and germination patterns were previously analyzed in two halophyte species, *Salicornia ramosissima* and *Atriplex halimus*. The different Ni concentrations hindered seedling development and limited plant colonization, thus affecting the phytoremediation process (Marquez-Garcia et al., 2013). In the present study, the seedling size and germination were also affected by different Ni concentrations (**Figure 3b**). Compared with the controls in this research, the FW, DW, relative root viability, and relative water content (RWC) were not significantly different when the Ni concentration was > 1.5 mM, and the relative root viability was > 65% with their RWC reaching > 80% (**Figures 3c, e**). Therefore, *H. glomeratus* has strong ability to tolerate Ni stress and the roles of Ni tolerance may sustain the higher relative root viability and RWC to maintain a positive water balance. These characteristics are similar to those previously reported for salt tolerance and Cd^2+^ tolerance of *H. glomeratus* (Yao et al., 2022; Wang et al., 2015). Together, these physiological results confirmed *H. glomeratus* can accumulate a significant amount of Ni^2+^ from root to leaf as well as sustain relative root viability and RWC to tolerate Ni stress, which may be valuable in phytoremediation of the Ni^2+^-contaminated environment. In addition, the size of water-storage tissue in leaf, the thickness of cortex in stem and the number of large parenchyma cells in root elevated in *H. glomeratus* with the increasing of Ni^2+^ concentrations (**Figures 4** a-i), but the Ni crystals in these tissue parts were not found (**Figures 5** a-f and **Figures 6** a-i), further study is needed in this area.

Proteomics not only served as a powerful tool for illustrating complete protein changes in organisms but also was used to compare variation in protein profiles at cell and organelle, tissue, and organ levels under different heavy metal stress (Ahsan et al., 2009). In *Phytolacca americana*, 14 proteins were enhanced expressed, and 11 reduced under Cd treatment, major changes were in photosynthetic pathway, GSH metabolism, transcription, translation and chaperones, 2 cys-peroxiadse and oxido-reductases proteins (Zhao et al., 2011). A large number of proteins involved in carbon metabolism showed a decrease in abundance, while proteins involved in remobilizing carbon from other energy sources were up-regulated in *Populus* sp. under Cd stress (Kieffer et al., 2008). The overexpressed proteins like oxygen-evolving enhancer protein, rubisco small subunit 1, chaperones, Fe-SOD, Mn-SOD, and heat shock like proteins were identified in *Chlamydomonas reinhardtii* under As stress, and RuBisCO large subunit and chloroplast 29kDa ribonucleoproteins were decreased for Oryza sativa (Walliwalagedara et al., 2012; Ahsan et al., 2010). Changes RuBisCO, defense/stress-related proteins, like the pathogenesis related class 5 protein (OsPR5), the probenazole-inducible protein (referred to as the OsPR10), and SOD were found in *Oryza sativa* under Cu stress (Hajduch et al., 2001). There were a lot of proteins were considered as the membrane proteins, such IRT1, an iron and zinc transporter, and FRO2, a ferric-chelate reductase, increased greatly in response to excess Zn (Fukao et al., 2011). A range of proteins differentially expressed in response to Mn containing a putative inorganic pyrophosphatase, a probenazole-inducible protein (PBZ1), a protein belonging to a universal stress protein (Usp) family, a chloroplast translational elongation factor (Tu) and the 50S ribosomal protein L1 (Führs et al., 2010). The significant biological processes and related differentially abundant proteins regulated the adaptation of *H. glomeratus* exposed to Cd^2+^ stress were also identified (Yao et al., 2022). However, the objective in the present study was to identify response mechanisms of *H. glomeratus* to Ni^2+^ stress using comparative proteomics. Xu et al. (2016) found differentially abundant proteins that helped elucidate the molecular mechanisms of heavy metal accumulation through *Amaranthus hybridus L.* roots (Haijun et al., 2016). A DIA-based proteomic method was performed to analyze the mechanisms of Cd^2+^ stress in *H. glomeratus* (Yao et al., 2022). In the present study, a greater number of increased abundant proteins were identified for *H. glomeratus* treated with Ni^2+^ stress, the functions of these proteins and their main pathways are discussed in the following study.

Heavy metal stress often affects the differentially abundant proteins associated with the biosynthesis processes located in the cell membrane, vacuolar membrane, and extracellular space to prevent the extended metal exposure of plant cells from ion leakage, membrane disintegration, DNA/RNA degradation, lipid peroxidation, and eventually death (Lin and Aarts, 2012). Plants integrate large amounts of processes in response to heavy metal stress, including the metabolic process, transmembrane transport, rRNA base methylation, cell recognition, and membrane organization (Henk et al., 2002). Proteins/genes involved in ion transmembrane transport play key roles in the response of *Vicia sativa* to heavy metal stress (Rui et al., 2018). We found that in *H. glomeratus* leaves, the biological process about “transmembrane transport”, “localization”, “establishment of location”, “phosphate ion transmembrane transport”, “inorganic anion transmembrane transport”, “RNA metabolic process”, “membrane organization” were significantly enriched at early stage of Ni^2+^ stress, which maybe regulate leaf cells of *H. glomeratus* to resist early Ni^2+^ stress. However, at the later stage of Ni^2+^ stress, a lot of biological processes related to survival ability of the plant cell to or resist the heavy metal stress and repair or replace damaged molecules were significantly enriched (**Figure 8**) (Nishikawa et al., 2000). Similar results have been observed in *Triticum aestivum* L (Pandolfini et al., 1992), *Thlaspi. goesingense* (Krmer et al., 1997), and *Thlaspi caerulescens* (Assuncao et al., 2010). Integrated cellular response to oxidative stress, hydrogen peroxide metabolic process, and cellular response to abscisic acid stimulus are particularly suited for analysis using proteomics (Finkel and Holbrook, 2000). In short, the changes observed in this study suggested that these biological processes at the early and later stages of Ni^2+^ stress in *H. glomeratus* may be of particular importance for Ni^2+^ tolerance in cells. We also focused on some proteins that are involved in the “Transport and catabolism” pathway. In the present study, A total of 15, 19, 18 and 23 proteins identified in this pathway in Ni^2+^-treated leaves at 6, 12, 24, and 48 h, respectively, which maybe play the well-known roles in Ni^2+^ hyperaccumulators for *H. glomeratus*, particularly members of the IREG and IRT/ZIP family. However, more researches are need to be performed to confirm the roles of these well known proteins in Ni^2+^ hyperaccumulation of *H. glomeratus.*

In addition, there were 86 significantly increased abundant proteins were commonly found at the four time points and mainly located in the apoplast (Haijun et al., 2016), plasma membrane (Rui et al., 2018), vacuolar membrane (Rui et al., 2018), chloroplast (Kim et al., 2004), and mitochondria (Mithofer, 2004) (**Figure 9**). Similar proteins were found in the hyperaccumulators Chinese flowering cabbage (Wang et al., 2017; Luo et al., 2017), maize line 178 (Shen et al., 2013), and radish roots under heavy metal stress environments (Yan et al., 2016). Halogeton_glomeratus_53126 protein showed highest increased abundance at the four time points (6, 12, 24, and 48 h) of Ni^2+^ stress and should be further investigated in future studies.

**Figure 10:**
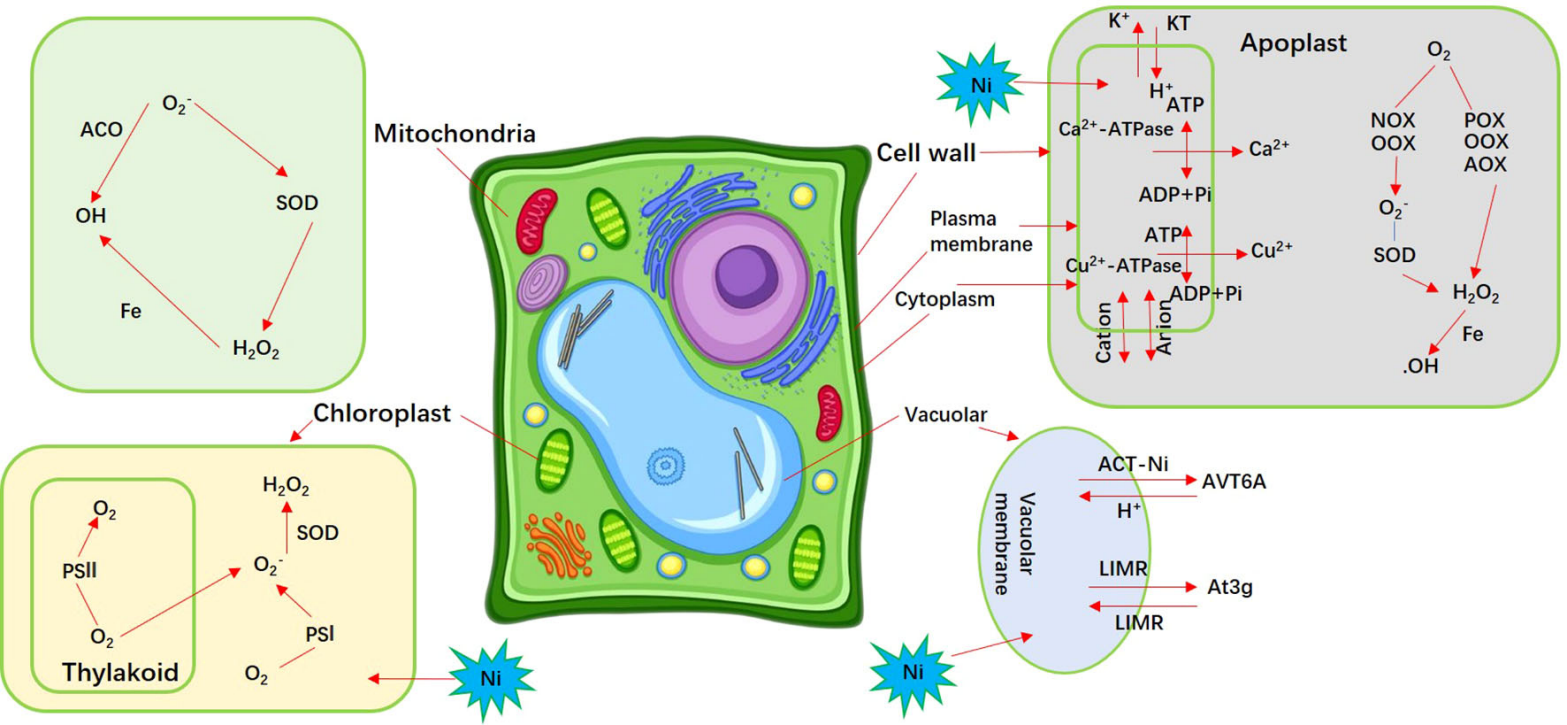
Detoxification mechanism for *H. glomeratus* in response to Ni^2+^ stress. ACT-Ni, an amino acid transporter; LIMR, LIMR family protein; KT, potassium transporter; Ca^2+^-ATPase, calcium-transporting ATPase; Cu^2+^-ATPase, copper-transporting ATPase; AOX, amine oxidase; OOX, oxalate oxidase; QOX quinone, oxidase; NOX, NADPH oxidase.

The toxicity of heavy metals in plants mainly inhibits chlorophyll synthesis, subsequently affecting photosynthesis and causing leaf chlorosis (Erbruggen et al., 2009). Under heavy metal stress, the Zn and Ca transport ion channels interacting with transport proteins affect protein structure and function, disrupt ion balance in plants, cause toxic symptoms such as nutritional imbalance, electron transfer system obstruction, stomata closure in plants, as well as excessive accumulation of ROS, leading to plant death (Qufei and Fashui, 2009; Lehotai et al., 2011; Maggio and Joly, 1995). The formation of chelating peptides enhances their clearance function while improving their ability to chelate or chelate heavy metals. The chelation products are more conducive to the transfer of heavy metal transporters to the cell wall and vacuoles (Barcelo et al., 1988; Henk and Sharmn, 1997). Ni hyperaccumulation in *Thlaspi goesingense* was primarily determined through its high Ni tolerance, achieved by an efficient system to pump and compartmentalize Ni in the vacuoles of shoot cells (Krmer et al., 1997; KrMer et al., 2000). An amino acid transporter (ACT-Ni) and LIMR family protein (LIMR) were highly abundant and located in the vacuolar membrane of *H. glomeratus* leaves, which may play a role in vacuolar sequestration of Ni in the hyperaccumulator *H. glomeratus* (Persans et al., 2001). Similar constitutively enhanced expression has also been observed for the TgMTP1 homologs AhMTP1 and ZTP1 in the Zn hyperaccumulators *Arabidopsis helleri* and *Thlaspi caerulescens* (Assuncao et al., 2010; Martina et al., 2004). Notably, the plant CDF family member TgMTP1 may also be acting at the plasma membrane as a metal efflux pump; a substantial amount of cellular Ni accumulates outside of the vacuole, indicating the need for a cytoplasmic-based Ni tolerance mechanism (Kim et al., 2004). Ni^2+^ mainly affected the H-ATPase activity and lipid composition of the plasma membrane in *Oryza sativa* shoots (Ros et al., 1992). We have identified and summarized KT (potassium transporter), calcium-transporting ATPase (Ca^2+^-ATPase), copper-transporting ATPase (Cu^2+^-ATPase), and transmembrane 9 superfamily member located in plasma membrane (**Figure 10**). In particular, heavy metal-absorbing proteins Zmys1, Zmysl2, and Osirt1 have been found in the plasma membrane and involved in the chelation of Ni and Ni^2+^-ATPase (Vert et al., 2021). COPT1–5 belongs to the high-affinity Cu transporter family with specific affinity for Cu^2+^ and responsible for the transport of Cu between organelles under heavy metal stress (Puig et al., 2014). Thus, these plasma membrane proteins related to transmembrane transport of ions and play important roles in detoxification of extravacuolar Ni in hyperaccumulating *H. glomeratus.* Furthermore, an uncharacterized membrane protein (Halogeton_glomeratus_42736) with increased abundance located in the plasma membrane, which was probably responsible for extravacuolar ions transport of *H. glomeratus* under Ni^2+^ stress.

The cell’s response to heavy metal stress includes changing antioxidant system in the plant clears ROS to protect cells from damage. Zhang et al. found the activities of SOD, CAT, and POX increased in rice treated with Cu and Pb (Zhang et al., 2014). In chloroplasts, ROS generated due to insufficient energy dissipation of PSII and PSII systems or light-induced charge recombination of electron pairs can generate H_2_O_2_ via the catalytic action of SOD (Tan et al., 2013). In mitochondria, the ROS generated by the reduction of electron carriers due to heavy metal stress can react to generate H_2_O_2_ via the catalytic action of SOD (Tan et al., 2013). We also found that hypothetical protein, CTP synthase isoform X1, CHD-type chromatin remodeling factor, putative nuclease and intron-binding protein aquarius with increased abundance located in the chloroplasts, and intron-binding protein aquarius, endoribonuclease/kinase IRE1, and NADH-nitrate reductase located in mitochondria of *H. glomeratus* leaves (**Figures 9**, **10**). It indicated that these proteins may be involved in regulating ROS antioxidant enzyme system synthesis to alleviate Ni toxicity for *H. glomeratus*. The apoplast between the plasma membrane and cell wall are the most active sites for ROS production and clearance. O_2_ in this site is generated through different pathways such as amine oxidase (AOX), oxalate oxidase (OOX), peroxidase, quinone oxidase (QOX), NADPH oxidase (NOX), and SOD (Hayward et al., 2013). Increased abundance of anion transporter 4, nucleobase-ascorbate transporter 6, and GASA-like protein GEG2 was observed in the apoplast (**Figures 9**, **10**). Obviously, high intracellular Ni^2+^ levels evidently induced changes in the abundance of proteins involved in ROS antioxidant enzyme system. Our DIA-based proteomic data cannot fully explain the current view of Ni^2+^ extrusion and hyperaccumulating in *H. glomeratus*. Further work is needed to better understand the mechanisms of Ni^2+^ regulation, including the integration of phenotypic, physiological and proteomics analysis, Ni^2+^ transport of the plasma membrane and the Ni^2+^ uptake system of the vacuolar, chloroplasts, mitochondria, and apoplast, based on both functional assays and proteomics of subcellular compartments containing plasma membrane and tonoplast proteomics analysis of *H. glomeratus* under Ni^2+^ stress.

In conclusion, *H. glomeratus* had robust resistance to Ni stress, can accumulate large amounts of Ni^2+^ in seedlings. Numerous differentially abundant proteins significantly enriched in biological processes and KEGG pathway play important roles in response and adaption to Ni^2+^ stress. In addition, several detoxification-related differentially abundant proteins involved in transmembrane transport and binding, which were mainly located in the tonoplast, plasma membrane, chloroplast, mitochondrion, and cytoplasm in *H. glomeratus* under Ni^2+^ stress, were identified. These results increased knowledge on the mechanisms of Ni^2+^ tolerance in phytoremediation plants and provided additional information for breeding new varieties with Ni tolerance for future application in agriculture.

## Data Availability

The raw data supporting the conclusions of this article will be made available by the authors, without undue reservation.

## Supplementary material

Supplementary File 1 Detailed information on peptides.

Supplementary File 2 Detailed information on proteins.

Supplementary File 3 Distribution of protein/peptide number based on proteomics of *H. glomeratus* treated with different Ni^2+^ concentrations.

Supplementary File 4 Differentially expressed proteins based on proteomics of *H. glomeratus* treated with 1.5 mM Ni^2+^ for 6 h.

Supplementary File 5 Differentially expressed proteins based on proteomics of *H. glomeratus* treated with 1.5 mM Ni^2+^ for 12 h.

Supplementary File 6 Differentially expressed proteins based on proteomics of *H. glomeratus* treated with 1.5 mM Ni^2+^ for 24 h.

Supplementary File 7 Differentially expressed proteins based on proteomics of *H. glomeratus* treated with 1.5 mM Ni^2+^ for 48 h.

Supplementary File 8 Differentially expressed proteins involved in primary biological processes of *H. glomeratus* treated with 1.5 mM Ni^2+^ for 6 h based on the GO classification.

Supplementary File 9 Differentially expressed proteins involved in primary biological processes of *H. glomeratus* treated with 1.5 mM Ni^2+^ for 12 h based on the GO classification.

Supplementary File 10 Differentially expressed proteins involved in primary biological processes of *H. glomeratus* treated with 1.5 mM Ni^2+^ for 24 h based on the GO classification.

Supplementary File 11 Differentially expressed proteins involved in primary biological processes of *H. glomeratus* treated with 1.5 mM Ni^2+^ for 48 h based on the GO classification.

Supplementary File 12 KEGG pathway analysis of differentially abundant proteins of *H. glomeratus* under 1.5 mM Ni^2+^ treatment for 6 h, 12 h, 24 h and 48 h.

Supplementary File 13 KEGG pathway annotation for the differentially abundant proteins of *H. glomeratus* under 1.5 mM Ni^2+^ treatment for 6 h.

Supplementary File 14 KEGG pathway annotation for the differentially abundant proteins of *H. glomeratus* under 1.5 mM Ni^2+^ treatment for 12 h.

Supplementary File 15 KEGG pathway annotation for the differentially abundant proteins of *H. glomeratus* under 1.5 mM Ni^2+^ treatment for 24 h.

Supplementary File 16 KEGG pathway annotation for the differentially abundant proteins of *H. glomeratus* under 1.5 mM Ni^2+^ treatment for 48 h.

Supplementary File 17 Differentially highly abundant proteins commonly found in *H. glomeratus* treated with 1.5 mM Ni^2+^ for 6, 12, 24, and 48 h.

Supplementary File 18 Differentially expressed proteins identified in *H. glomeratus* involved in transporting Ni^2+^ and Ni-chelate under Ni stress.
