# Supplementary material for: Physiological and proteomic analysis of halophyte Halogeton glomeratus in response to Ni2+ stress
Source: Front Plant Sci. 2026 Jan 30;16:1622321. doi: 10.3389/fpls.2025.1622321 (PMC12903123; doi:10.3389/fpls.2025.1622321)
Supplement: Supplementary File 1 — Detailed information on peptides. [file DataSheet1.zip › supplementary file 12.docx]

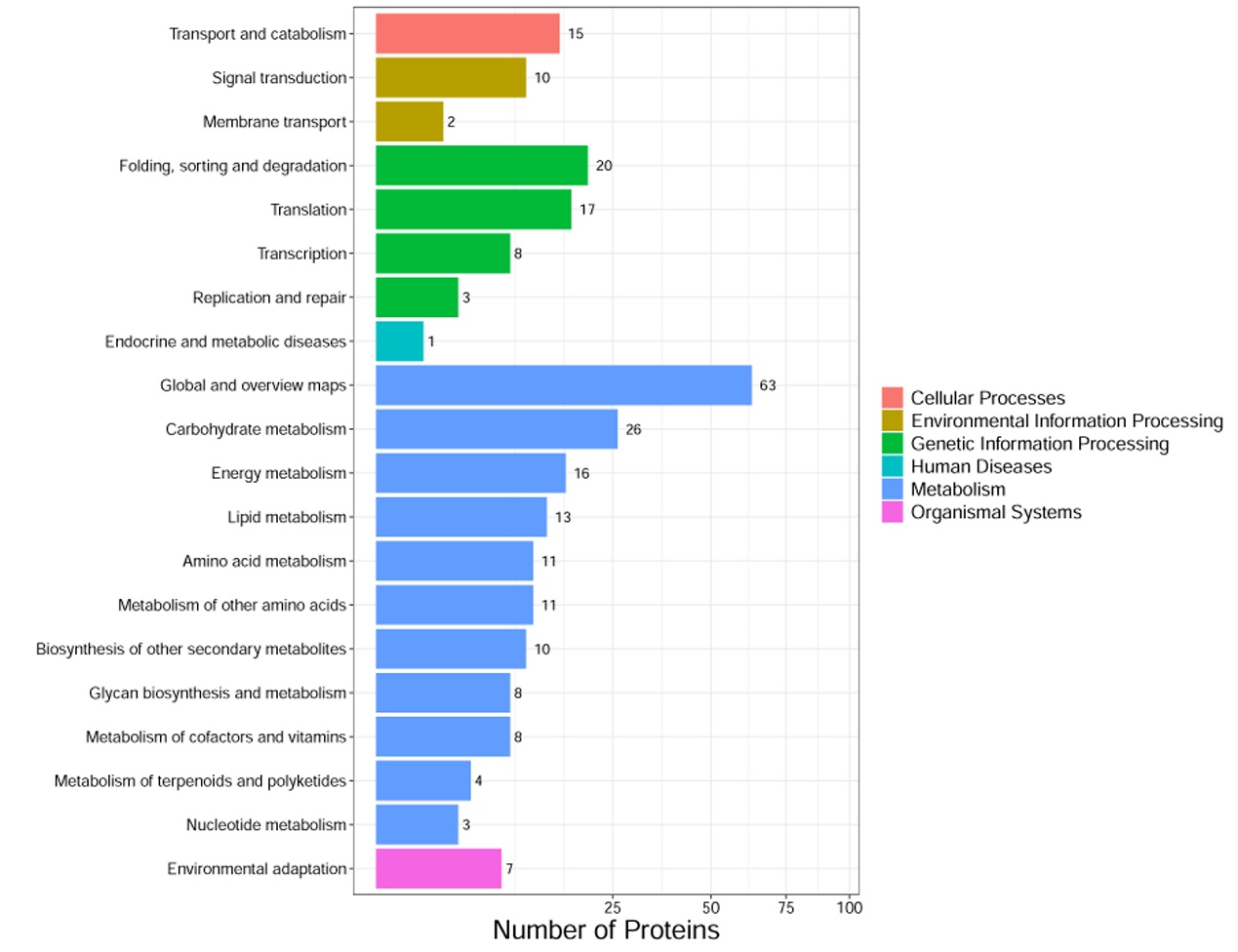


Supplementary File 11. KEGG pathway analysis of differentially abundant proteins of *H. glomeratus* under 1.5 mM Ni^2+^ treatment for 6 h, 12 h, 24 h and 48 h.


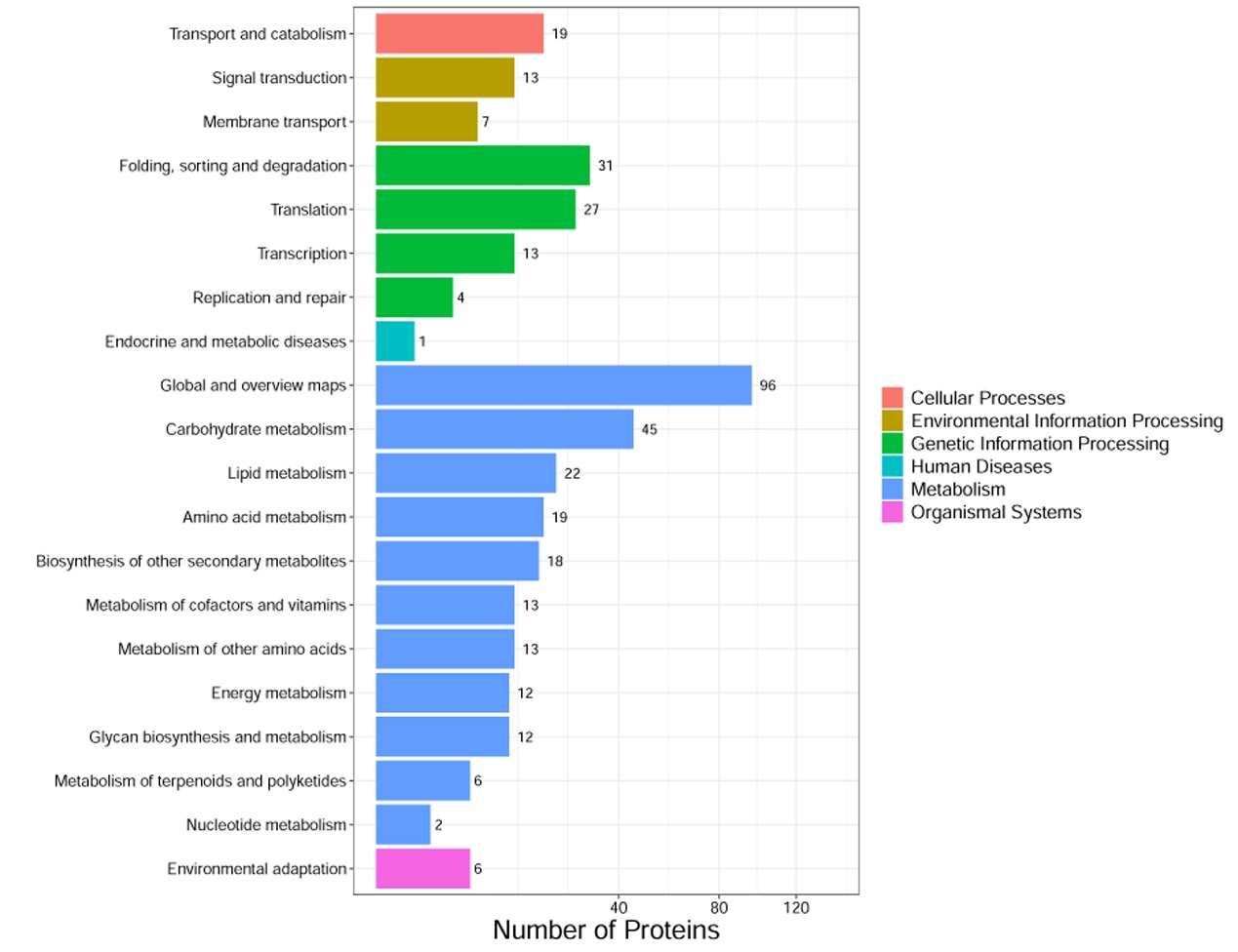


**Figure 2** KEGG pathway analysis of differentially abundant proteins of *H. glomeratus* under 1.5 mM Ni^2+^ treatment for 12 h.


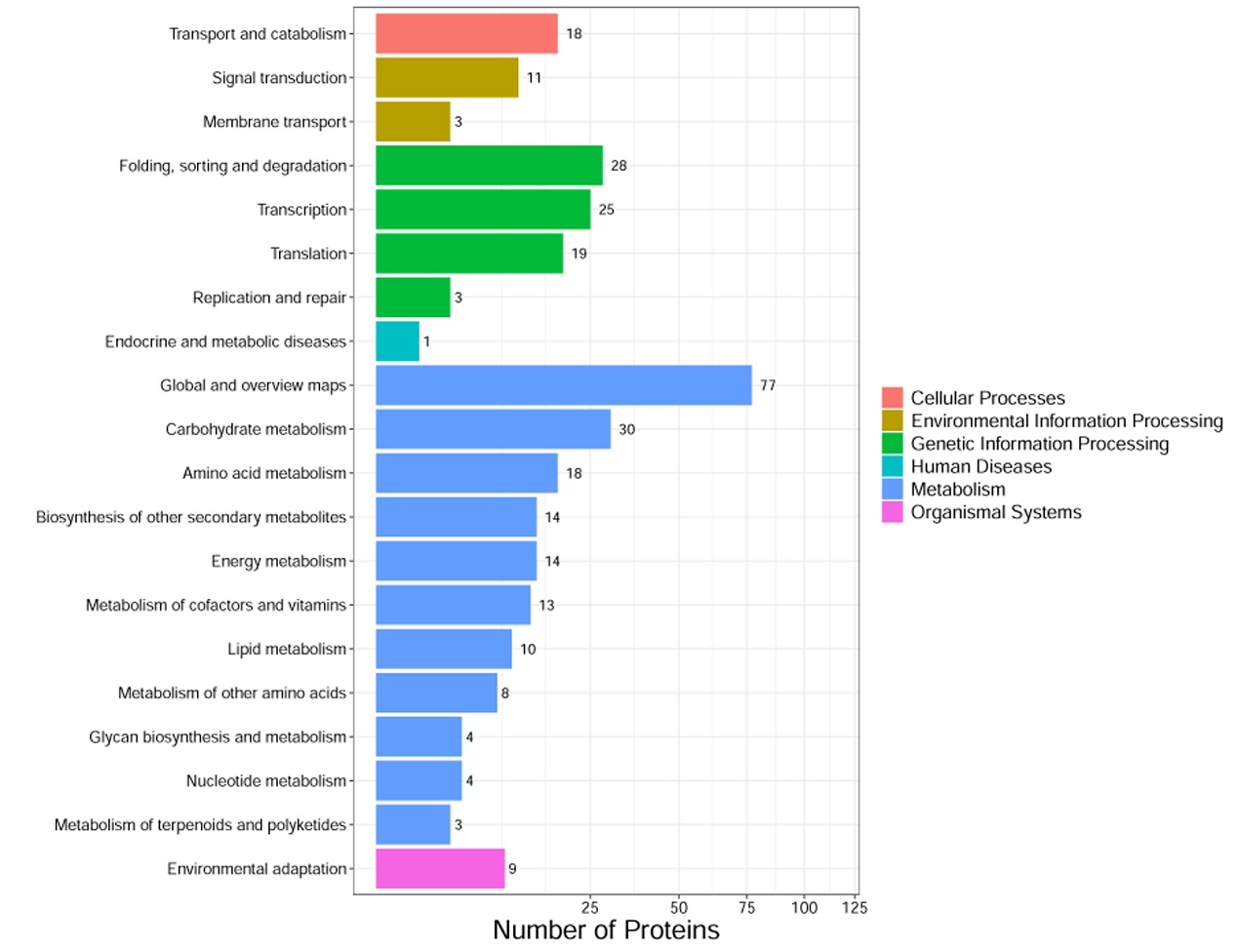


**Figure 3** KEGG pathway analysis of differentially abundant proteins of *H. glomeratus* under 1.5 mM Ni^2+^ treatment for 24 h.


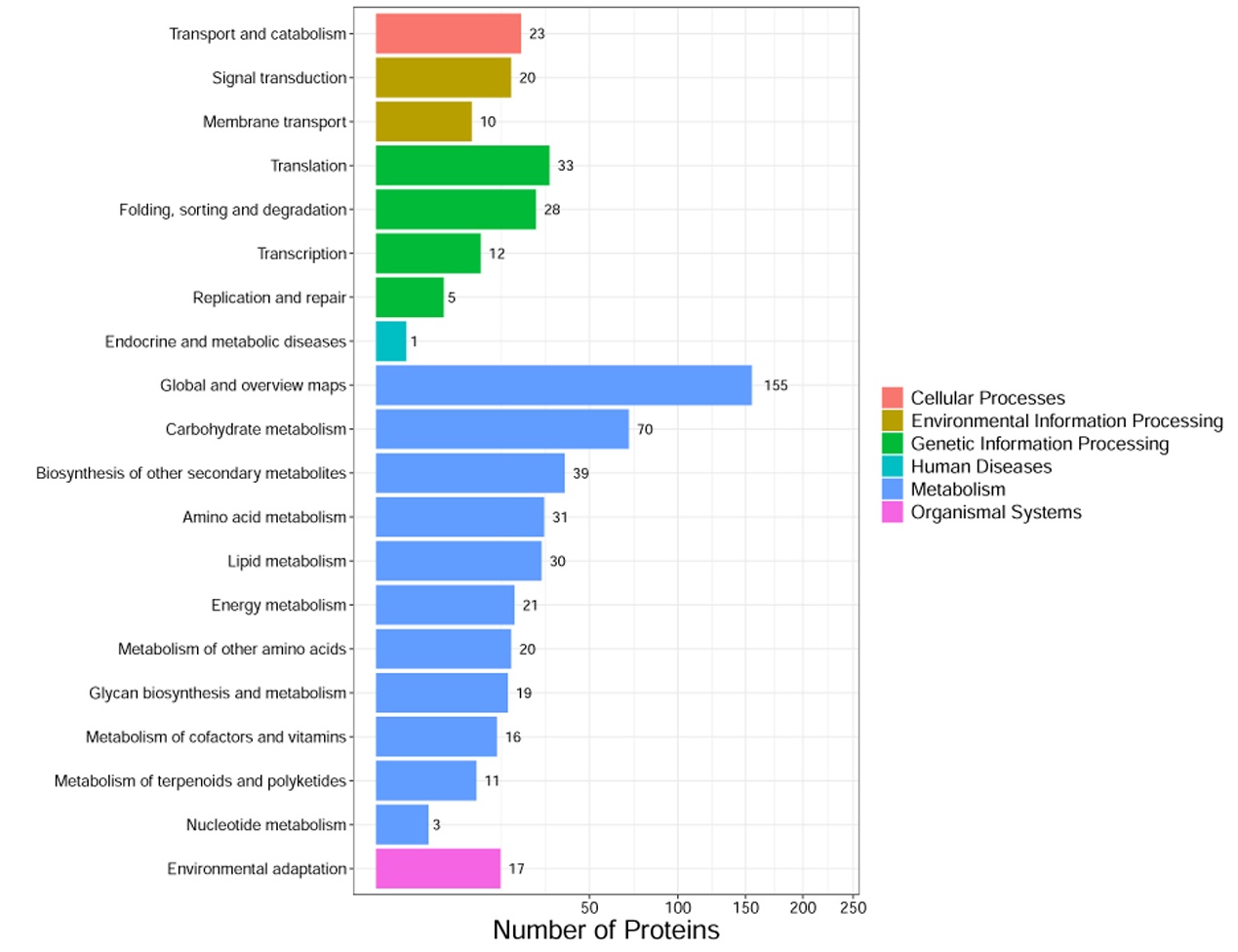


**Figure 4** KEGG pathway analysis of differentially abundant proteins of *H. glomeratus* under 1.5 mM Ni^2+^ treatment for 48 h.
