# Supplementary figures and images for: Physiological and proteomic analysis of halophyte Halogeton glomeratus in response to Ni2+ stress
Source: Front Plant Sci. 2026 Jan 30;16:1622321. doi: 10.3389/fpls.2025.1622321 (PMC12903123; doi:10.3389/fpls.2025.1622321)

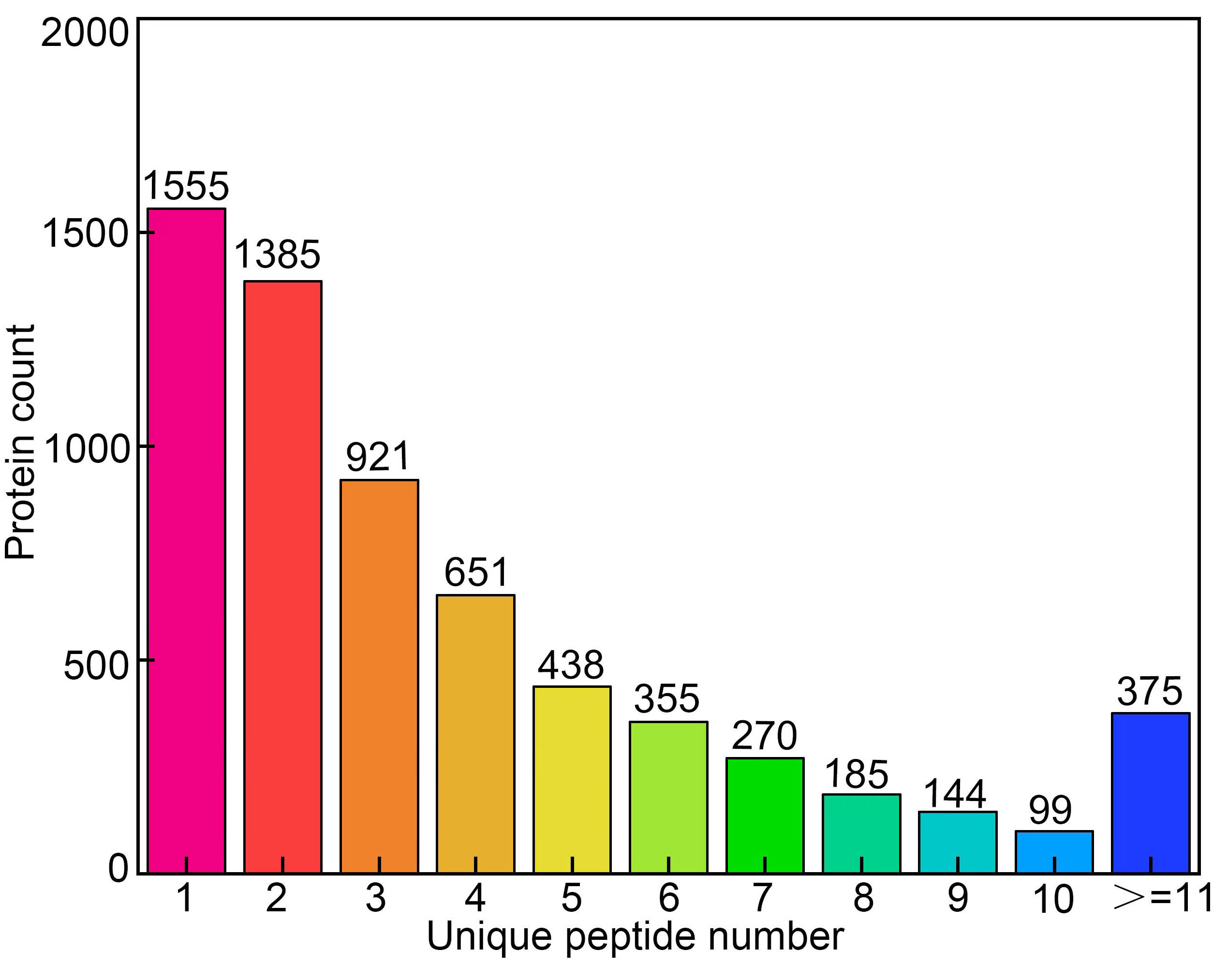

Supplement: Supplementary File 1 — Detailed information on peptides. [file DataSheet1.zip › supplementary file 3.jpg]
